# Cellular and Network Mechanisms May Generate Sparse Coding of Sequential Object Encounters in Hippocampal-Like Circuits

**DOI:** 10.1523/ENEURO.0108-19.2019

**Published:** 2019-08-19

**Authors:** Anh-Tuan Trinh, Stephen E. Clarke, Erik Harvey-Girard, Leonard Maler

**Affiliations:** 1 Department of Cellular and Molecular Medicine, University of Ottawa, Ottawa, Ontario K1H 8M5, Canada; 2 Brain and Mind Institute, Center for Neural Dynamics, University of Ottawa, Ottawa, Ontario K1H 8M5, Canada; 3 Department of Bioengineering, Stanford University, Stanford, CA 94305

**Keywords:** hippocampus-like, recurrent network, sparse coding, spike threshold adaptation, time cell, weakly electric fish

## Abstract

The localization of distinct landmarks plays a crucial role in encoding new spatial memories. In mammals, this function is performed by hippocampal neurons that sparsely encode an animal’s location relative to surrounding objects. Similarly, the dorsolateral pallium (DL) is essential for spatial learning in teleost fish. The DL of weakly electric gymnotiform fish receives both electrosensory and visual input from the preglomerular nucleus (PG), which has been hypothesized to encode the temporal sequence of electrosensory or visual landmark/food encounters. Here, we show that DL neurons in the *Apteronotid* fish and in the *Carassius auratus* (goldfish) have a hyperpolarized resting membrane potential (RMP) combined with a high and dynamic spike threshold that increases following each spike. Current-evoked spikes in DL cells are followed by a strong small-conductance calcium-activated potassium channel (SK)-mediated after-hyperpolarizing potential (AHP). Together, these properties prevent high frequency and continuous spiking. The resulting sparseness of discharge and dynamic threshold suggest that DL neurons meet theoretical requirements for generating spatial memory engrams by decoding the landmark/food encounter sequences encoded by PG neurons. Thus, DL neurons in teleost fish may provide a promising, simple system to study the core cell and network mechanisms underlying spatial memory.

## Significance Statement

To our knowledge, this is first study of the intrinsic physiology of teleost pallial (DL) neurons. Their biophysical properties demonstrate that DL neurons are sparse coders with a dynamic spike threshold leading us to suggest that they can transform time-stamped input into spatial location during navigation. The concept of local attractors (bumps) that potentially move “across” local recurrent networks has been prominent in the neuroscience theory literature. We propose that the relatively simple and experimentally accessible DL of teleosts may be the best preparation to examine this idea experimentally and to investigate the properties of local (excitatory) recurrent networks whose cells are endowed with, e.g., slow spike threshold adaptation dynamics.

## Introduction

The mammalian hippocampus is required for the storage and recall of spatial memory that presumably guides path integration and landmark based navigation (Barry and Burgess, 2014; Hartley et al., 2014). Conventionally, sparse discharge of dentate gyrus (DG) granule cells and CA1/CA3 pyramidal cells can encode a rodent’s location with respect to visually identified landmarks (Barry and Burgess, 2014; Hartley et al., 2014). An emerging alternate view of hippocampal function emphasizes its role in the encoding of temporal sequences within or across periods of locomotion (Pastalkova et al., 2008; MacDonald et al., 2011; Kraus et al., 2013; Eichenbaum, 2014; Modi et al., 2014; Ranganath and Hsieh, 2016). For example, hippocampal neurons may discharge at specific times after the initiation of running and effectively tile an entire running episode (Kraus et al., 2013). The encoding of time and location appears to be closely connected with the responses of a subset of neurons to time spent and distance traveled (Kraus et al., 2013; Deuker et al., 2016; Eichenbaum, 2017).

Visuospatial memory is also important for teleost fish (Rodríguez et al., 2002), and they can learn to finely discriminate between visual inputs (Schluessel and Bleckmann, 2005; Siebeck et al., 2009; Rischawy and Schuster, 2013; Newport et al., 2016). Unlike mammals, fish do not have an obvious cortex or hippocampus; instead, their dorsal telencephalon (pallium) is divided into non-layered cell groups that have specific connectivity and function (Rodríguez et al., 2002; Northcutt, 2008; Giassi et al., 2012b,c). Visual input to the pallium primarily arrives from the optic tectum and reaches the dorsolateral pallium (DL) through the thalamus-like preglomerular nucleus (PG; Yamamoto and Ito, 2008; Giassi et al., 2012b; Wallach et al., 2018). Lesion studies have shown that DL is essential for visual (landmark) based spatial learning and memory (Rodríguez et al., 2002).

Comparisons of teleost pallium to mammalian dorsal telencephalon has been controversial, and similarity between DL and either hippocampus or cortex have been stressed. Based on its location (Yamamoto et al., 2007; Mueller and Wullimann, 2009), extrinsic connections (Elliott et al., 2017), and molecular markers (Harvey-Girard et al., 2012; Ganz et al., 2014), it has been proposed that DL is homologous to the hippocampus (in particular to DG; Elliott et al., 2017). However, unlike the major recipients of sensory information in the hippocampal formation (i.e., DG, CA1), DL neurons have strong local recurrent connectivity (Trinh et al., 2016). DL’s extrinsic and intrinsic connectivity also suggests a strong resemblance to the mammalian cortex (Yamamoto et al., 2007; Giassi et al., 2012b; Trinh et al., 2016; Elliott et al., 2017). However, DL neurons are morphologically very different from both DG granule cells and the pyramidal cells of the hippocampus and cortex (Giassi et al., 2012c).

A teleost subgroup, the weakly electric gymnotiform fish, can use their electrosensory system to finely discriminate temporal (Harvey-Girard et al., 2010) and spatial (Graff et al., 2004; Dangelmayer et al., 2016) patterns and use electrosensory-identified landmarks to learn the spatial location of food (Jun et al., 2016). Electrosensory input is first processed in the hindbrain electrosensory lobe (ELL) and, via a midbrain relay, then mapped onto the tectum (Krahe and Maler, 2014). Electrosensory and visual tectal cells then project to PG and their PG target then projects exclusively to DL (Giassi et al., 2012b). Two recent studies have shown that DL cells can process visual and electrosensory inputs. In goldfish, Vinepinsky et al. (2018) have described DL cells responsive to boundaries (visual input) as well as speed and direction of self-motion. In a gymnotiform fish, neurons within a major target of DL (dorsal pallium, DD) have been shown to discharge to the electrosensory signals generated when the fish moves near “landmarks” (Fotowat et al., 2019).

Recently, a subset of electrosensory motion PG neurons have been identified that can encode the time interval between object encounters (Wallach et al., 2018). Wallach et al., hypothesize that the output of these “time stamp” neurons is used to estimate the distance between the objects encountered by the fish, thereby supporting the observed electrosense-dependent spatial learning (Jun et al., 2016). Given the similar anatomic and functional organization of visual and electrosensory motion pathways, we hypothesize that the transformation of electrosensory motion signals to a spatial map are processed in DL. Here, we studied the biophysical properties of DL neurons *in vitro* to determine if their intrinsic properties are compatible with their putative role in converting temporal input from PG (i.e., time between object encounters) to a spatial map (Wallach et al., 2018).

## Materials and Methods

For the following experiments, we used two closely related *Apteronotid* fish of either sex (*Apteronotus leptorhynchus* and *Apteronotus albifrons*), a suborder of the gymnotiform family, as well as *Carassius auratus* (goldfish) of either sex. The brains of *A. leptorhynchus* and *A. albifrons* cannot be readily distinguished; these species have been used interchangeably in previous anatomic studies (Carr et al., 1982) and the processing of electrosensory input appears to be nearly identical in these species (Martinez et al., 2016). Goldfish were included in this study for three reasons. First, we found that *Apteronotus* DL cells were challenging to maintain in slice preparation, whereas goldfish DL cells were more robust, yielding higher success rates on our lengthier protocols involving pharmacological manipulations. Second, we wanted to check how our results generalized to non-electrosensory teleosts, given the very general mechanisms of sparse neural coding proposed in this article. Last, the critical behavioral experiments on the essential role of DL in spatial memory were done in goldfish (Rodríguez et al., 2002), setting a precedent in the literature; further, the first *in vivo* DL recordings have also been conducted in goldfish (Vinepinsky et al., 2018). As demonstrated in the results, our conclusions apply equally well to each of these species and are therefore directly relevant to spatial learning across a broad range of teleost fish.

Before use, the *Apteronotus* fish were kept in heated aquariums at 28°C, while goldfish were kept in aquariums at 22°C (room temperature). All procedures were approved by the University of Ottawa Animal Care Committee and follow the guidelines issued by the Society for Neuroscience.

### Slice preparation

Before the dissection, adult male and female fishes were anesthetized in oxygenated water containing 0.2% 3-aminobenzoic ethyl ester (tricaine methanesulfonate, Aqua Life, Syndel Laboratories). As the skull was being removed, ice cold oxygenated (95% O_2_, 5% CO_2_) artificial CSF (ACSF; 130 mM NaCl, 24 mM NaHCO_3_, 10 mM glucose, 2.5 mM KCl, 1.75 mM KH_2_HPO_4_, 1.5 mM CaCl_2_, 1.5 mM MgSO_4_, and 295 mOsm, pH 7.4), containing 1 mM of kynurenic acid (Millipore Sigma), was dripped onto the fish’s brain. The brain was then carefully removed and submerged in a Petri dish containing ice-cold ACSF with kynurenic acid. Once the brain was removed, it was placed in an ice-cold cubic mold, to which oxygenated ACSF mixed with 2.5% low-melting agarose (Millipore Sigma) was added. After the agarose has solidified, an initial cut was performed to separate the telencephalon from the rest of the brain. Subsequently, 300-µm-thick transverse brain slices of the telencephalon were obtained using a vibratome. For goldfish dissections, a slightly different cutting ACSF was used: 108 mM NaCl, 24 mM NaHCO_3_, 10 mM glucose, 2.5 mM KCl, 1.25 mM KH_2_HPO_4_, 1.5 mM CaCl_2_, 1.5 mM MgSO_4_, and 2 mM HEPES, 260 mOsm (adapted from Palmer, 2006). Furthermore, the thick optic nerves underneath the brain had to be severed with micro scissors before the brain was removed and placed in a Petri dish containing ice-cold ACSF. The rest of the dissection was done in the same manner as in *Apteronotus* (see Trinh et al., 2016). Brain slices containing the dorsolateral telencephalon (DL) were then transferred into a continuously oxygenated slice incubation chamber containing ACSF where they were left to rest for 30–60 min.

### *In vitro* recordings

**Figure 1.**
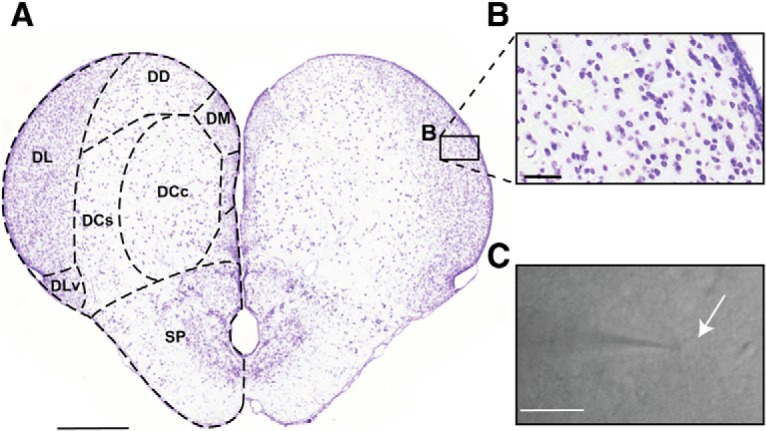
Anatomy of the *A. leptorhynchus* telencephalon. ***A***, A transverse section through the *Apteronotus* telencephalon indicating the major subdivisions of pallium and subpallium (SP); this section was obtained from a standard series of cresyl violet-stained sections (Elliott et al., 2017). Midbrain sensory inputs entering the pallium from PG terminates in the DL. These inputs are processed within the DL recurrent network (Trinh et al., 2016). DL projects to the core dorsocentral pallium (DCc) which, in turn, projects to midbrain sensory regions. DL, ventral subdivision (DLv) is located ventral to DL and distinguished by its olfactory bulb input. The dorsal-dorsal pallium (DD) has reciprocal connections with DL (Elliott et al., 2017). Scale bar: 500 µm. ***B***, A higher magnification of the cells in DL illustrates an apparent random distribution and its highly organized intrinsic laminar and columnar circuitry is not evident (Trinh et al., 2016). The neurons in DL have homogenous morphology and are roughly 10 µm in diameter (Giassi et al., 2012c). Scale bar: 50 µm. ***C***, An infrared image of a DL neuron undergoing a whole-cell patch recording. The shadow to the left illustrates the patch pipette, while the white arrow highlights the patched cell. Scale bar: 20 µm. DCs, dorsocentral pallium, shell; DM, dorsomedial pallium.

**Figure 2.**
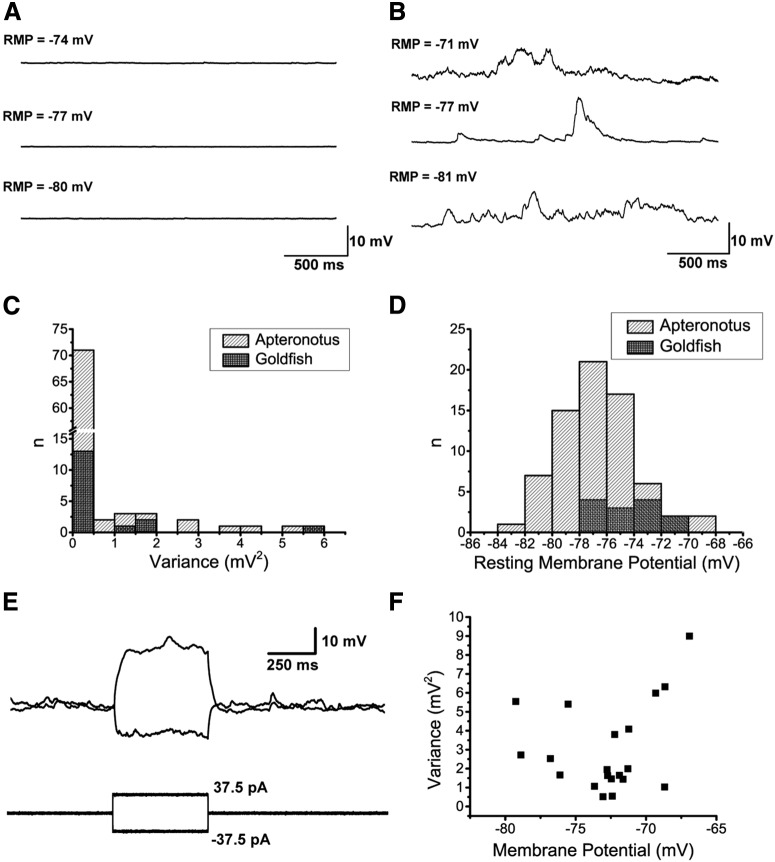
RMP of DL neurons. ***A***, Three example RMP traces taken from three different quiet neurons illustrate the membrane potential at which these cells would normally stabilize at naturally (i.e., no holding current was applied). The RMP at the start of the recording is shown above each trace. ***B***, Three example RMP traces taken from three different noisy neurons in which no holding current was applied. In contrast to the quiet cells, these cells exhibited strong membrane fluctuations even when they had stabilized at a hyperpolarized potential. ***C***, A histogram of the RMP variance for *Apteronotus* (gray) and goldfish (black) DL neurons showing that most neurons were of the quiet type where *n* is the number of individual 10-s recording traces that were recorded from all cells (*Apteronotus*, *N* = 29 cells; goldfish, *N* = 7 cells; total *n* = 85 recordings). ***D***, A histogram of the natural RMPs in both the *Apteronotus* and in the goldfish illustrating that the average RMP of DL neurons is around –77 mV in *Apteronotus* and around –73 mV in goldfish (*Apteronotus*, *N* = 35 cells; goldfish, *N* = 11 cells; total *n* = 71 recordings). ***E***, A noisy DL neuron’s response to the injection of ±37.5-pA current steps in *Apteronotus*, illustrating that the membrane fluctuations are invariant to the membrane potential of the cell. ***F***, A scatter plot of the variance and membrane potential, including all recordings (black dots) that had a variance value above 0.5 mV^2^ (*Apteronotus*, *N* = 6 cells; goldfish; *N* = 4 cells; total *n* = 20 recordings).

After the incubation period, brain slices containing DL were transferred to the recording chamber where oxygenated ACSF was constantly perfused at a flow rate of 3 ml/min. Recordings were performed at room temperature (23–24°C). We used fire-polished borosilicate glass micropipettes (Sutter Instruments) with resistances ranging between 8 and 14 MΩ. The intracellular solution contained the following: 130 mM K-gluconate, 10 mM KCl, 10 mM HEPES, 4 mM NaCl, 4 mM Mg-ATP, 10 mM phosphocreatine, and 0.3 mM Na-GTP, with an osmolality of 295 mOsm, and a pH of 7.2 for weakly electric fish recordings. A silver wire plated with silver chloride was used as a ground. For goldfish experiments, recordings were done in the goldfish ACSF as described above and a slightly different intracellular solutions was used: 110 mM K-gluconate, 10 mM KCl, 18 mM HEPES, 4 mM Mg-ATP, 10 mM phosphocreatine, and 0.3 mM Na-GTP, 265 mOsm, pH 7.2. To visualize the neurons, slices were imaged under differential interference contrast (DIC) optics using a CMOS infrared camera (Scientifica) directly connected to the rig computer (Fig. 1). The recording signals were amplified using a Multiclamp 700B (Molecular Devices), while the signal was filtered at 3 kHz and digitized using a Digidata 1550 (Molecular Devices). The whole-cell recording data were acquired using the PClamp 10.6 software (Molecular Devices, RRID: SCR_011323). All recordings were performed in current-clamp mode. Only cells that required a minimal holding current less than –50 pA were included in the study, allowing to stabilize the cell near the average resting membrane potential (RMP; ∼–75 mV; Fig. 2*E*). The maximal recording time after the dissection was 4–5 h. Once the whole-cell configuration was obtained, the RMP was recorded for 10 s, and the cells were injected with current steps, which typically range from 500 to 1000 ms and from –60 to +60 pA, except where otherwise noted. For our ramp current protocol, we injected two different ramp currents at different inter-stimulus time intervals ranging from 50 to 1000 ms. Although both ramp stimuli have the same slope, the first ramp current was always two-fold stronger than the second ramp since the first ramp current had to evoke multiple action potentials while the second one only had to evoke one action potential. As such, the magnitude of the second current injection had to be adjusted for each cell since the rheobase for each cell is different and the magnitude of the first ramp was then adjusted according to the second ramp. Healthy cells were usually held for 30–60 min.

### Pharmacology

A subset of DL cells exhibited membrane “noise.” We bath applied the non-selective antagonist, kynurenic acid (10 mM; Millipore Sigma), to block ionotropic glutamatergic transmission to determine if this noise was due to synaptic input to DL cells.

To test for the presence of fast and persistent sodium channels in DL neurons (Berman et al. 2001), we first patched the cell and injected a standard 500-ms current step before applying 20 µM tetrodotoxin (TTX; Abcam) locally near the recording site by pressure injection. To further investigate the presence of a persistent sodium channel, we also applied 5 mM lidocaine N-ethyl bromide (QX-314; Millipore Sigma) via the intracellular recording solution to block sodium (Salazar et al., 1996) and other channels (e.g., certain K^+^ channels and Ca^2+^ channels, see Results; Alreja and Aghajanian, 1994; Perkins and Wong, 1995; Talbot and Sayer, 1996).

Calcium-activated potassium channels SK1/2 are both expressed in DL (Ellis et al., 2008). We used our standard current step protocol to evoke spikes in patched DL cells and bath applied an SK channel blocker 30 µM 6,12,19,20,25,26-hexahydro-5,27:13,18:21,24-trietheno-11,7-metheno-7*H*-dibenzo [*b*,*n*] [1,5,12,16] tetraazacyclotricosine-5,13-diium dibromide (UCL; Tocris, Bio-Techne). We also locally applied 1 mM 1-ethyl-2-benzimidazolinone (EBIO; Abcam), a SK channel agonist near the brain slice by pressure injection. Finally, we patched neurons using a slightly altered internal solution that contained 10 mM BAPTA (Millipore Sigma) to chelate intracellular calcium. The osmolarity of this intracellular solution was readjusted to 295 mOsm.

### RT-PCR

G-protein-coupled inwardly-rectifying potassium channels (GIRK) 1–4 mRNA sequences were identified from *A. leptorhynchus* brain transcriptome data (Salisbury et al., 2015). Two degenerate PCR primers were designed to bind all GIRK isoform sequences (forward: CTGGTGGACCTSAAGTGGMG; reverse: TTCTTGGGCTGNGNAGATCTT). Five *A. leptorhynchus* fish were anesthetized with tricaine methanesulfonate (Aqua Life, Syndel Laboratories) and then sacrificed by cervical dislocation while being fed oxygenated water containing the anesthetic. Different regions of the brain (DL, tectum/torus, subpallium, cerebellum, ELL, hindbrain) were dissected in ice-cold ACSF, collected and preserved on dry ice. All tissues were weighed, and homogenized in Trizol to purify total RNA (Millipore Sigma). First-strand cDNAs were then generated by using the RevertAid H Minus First Strand cDNA Synthesis kit (Fermentas). Degenerate PCR was performed using the DreamTaq, according to the manufacturer recommendations (Thermo Fisher Scientific), with the primers mentioned above. On an agarose gel, the amplicon expected bands were 344 bp.

### Data analysis

**Figure 3.**
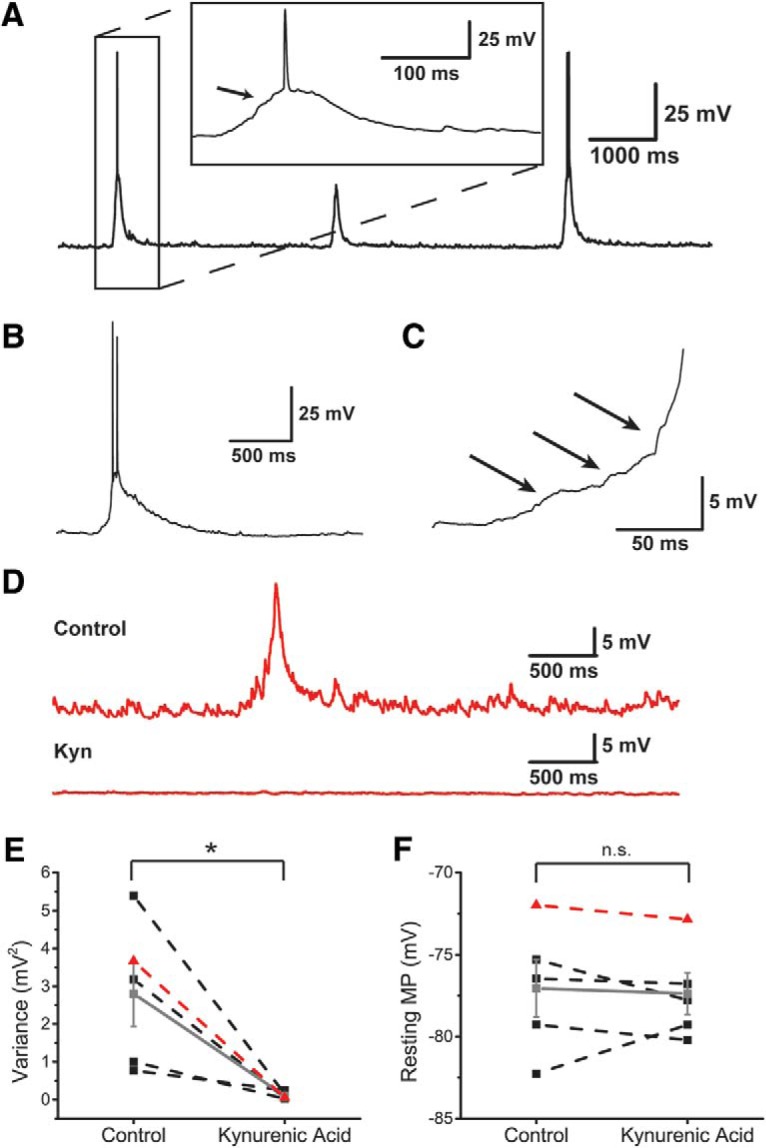
Noisy cells. ***A***, Example recording trace from a noisy cell displaying spontaneous membrane potential fluctuations. These fluctuations often vary in size but are usually in the range of several millivolts and can trigger action potentials (spikes), as highlighted by the box showing a magnified version of the first fluctuation. The arrow within the magnified box highlights an example of the small fluctuations that precede spiking. ***B***, Example trace illustrating a spontaneous membrane fluctuation that lasted 865.5 ms and produced a short burst of 2 action potentials. ***C***, A higher magnification of the rise phase of the spontaneous fluctuation shown in panel ***B***. The arrows denote small membrane potential fluctuations that appear to summate, giving rise to a sustained depolarization and spiking. ***D***, The top trace illustrates an example recording of a noisy cell before the addition of a synaptic blocker. The bottom trace illustrates a recording of the same cell after the addition of 10 mM kynurenic acid. ***E***, The average variance of the RMP before and after the bath application of 10 mM kynurenic acid (*Apteronotus*; *N* = 3 cells, goldfish; *N* = 2 cells). Each black square represents a cell from either fish and the gray square represents the mean variance. The red triangles depict the average variance of the cell shown in ***D***. Of particular note, the wide range of variances all decreased to a similar value after the application of the synaptic blocker. ***F***, Same as in ***E*** except this graph depicts the RMP instead (*Apteronotus*; *N* = 3 cells, goldfish; *N* = 2 cells). Unlike the variance, the RMP was unaffected by the bath application of the synaptic blocker. *p < 0.05. n.s, not significant.

All the recording data were first visualized in Clampfit (Molecular Devices) before being transferred into MATLAB (MathWorks, RRID: SCR_001622) for subsequent analysis with custom scripts. To reduce the likelihood of analyzing unhealthy cell responses, only cells which produced spikes that cross a data-driven threshold of –5 mV were included in the analysis. Cells that showed significant membrane noise, i.e., a variance >0.5 mV^2^, were used to construct Figures 2 and 3 but were excluded from any additional analysis. For the analysis of the RMP (Fig. 2), only cells that did not require a holding current to stabilize were included in this analysis. For the analysis of the average RMP (and variance) in Figure 3, a total of 2 min of recording (binned into 10-s sweeps) were analyzed for each cell before and after the addition of the synaptic blocker (for a total of 4 min per cell). If the recording trace contained any spontaneous action potentials, the action potentials were replaced with the membrane potential recorded in the prior 100 ms. The membrane time constant was measured by fitting an exponential function to the neuron’s recovery to equilibrium following injection of a negative step current. The spike amplitude was measured by two methods: first, as the difference between the spike height and the spike threshold and, second, from the difference between the spike height and the RMP. To estimate the spike threshold, we used the method of Azouz and Gray (2000) which defined the spike threshold as the voltage corresponding to an empirically defined fraction (0.033) of the peak of the first derivative. This first derivative method was later shown to be slightly better than the second derivative method (Sekerli et al., 2004) previously used for hindbrain electrosensory neurons (Chacron et al., 2007). The threshold for the broad Ca^2+^ spikes were determined visually in Clampfit since the rate of change of the Ca^2+^ spike was too slow to be visualize with either the first or second derivative of the membrane potential. The spike width was calculated by measuring the half-width at half-maximum. The voltage, as a function of injected current (I-V curves), was obtained in Clampfit using sub-threshold traces and averaged to reduce the variability across cells caused by the holding current. The input resistance was obtained by calculating the average slope of the I-V curve across all cells. The after-hyperpolarizing potential (AHP) amplitude was measured as the difference between the spike threshold and the minimum value of the AHPs. If the recording trace contained a burst or spike doublet, then the AHP would be measured on the following spike, since a doublet would typically induce an especially large AHP. The cell’s average firing rate was calculated as the number of spikes divided by the duration of the stimulus. The δ spike height was calculated as the difference in spike height between the *n*th spike and the first spike of an evoked spike train. The interspike interval (ISI) was measured as the time between the first two spikes of the spike train induced by a current step injection, while the δ time was calculated as the difference between the time of the first AHP and the time of the *n*th AHP. The δ AHP was obtained by subtracting the first spike’s AHP amplitude from the second spike’s AHP amplitude. The δ threshold was obtained in a similar fashion. All error bars were determined using the standard error of the mean. Wherever applicable, the statistical significance was determined using either one-way ANOVA, two-way ANOVA, one sample *t* test, two-sample *t* test or the paired *t* test, where *p* < 0.05 is considered significant.

### Inactivating exponential integrate and fire model (iEIF)

To illustrate the putative role of slow sodium channel inactivation on the observed and variable spike threshold in DL cells, we sought a minimal neuron model that incorporates an abstraction of sodium channel dynamics. The inactivating exponential integrate and fire neuron (Eq. 1; Platkiewicz and Brette, 2011) provides a distilled representation of sodium channel activation via an exponential amplification of the membrane voltage (*V*), which is attenuated by fast and slow inactivation variables (*h_f_* and *h_s_*). These sodium channel inactivation terms further affect the dynamic threshold for spike generation, *θ*, whose initial value *V_T_* reflects no inactivation at the RMP (Eqs. 2, 3; *h_f_* = *h_s_* = 1; Platkiewicz and Brette, 2010). Although the exponential approximation does not realistically capture the full action potential wave form, which spans a large voltage range, it is valid for voltages near spike initiation. Importantly, this approximation permits the differential equation for the variable spike threshold, *θ*, to be simply expressed by sodium channel properties described in Equations 2, 3 (Platkiewicz and Brette, 2010, 2011).

(1) $$\begin{array}{ll} C\frac{dV}{dt}=g_{L}h_{f}h_{s}e^{\frac{V-V_{T}}{k_{a}}}+g_{L}\left(E_{L}-V\right)+I & \text{for }V<V_{T}, \end{array}$$

(2) $$\theta =V_{T}-k_{a}\log(h_{f}h_{s}),$$

(3) $$V_{T}=V_{a}-k_{a}\log(\frac{g_{Na}}{g_{L}}\frac{E_{Na}-V_{a}}{k_{a}}).$$

**Figure 4.**
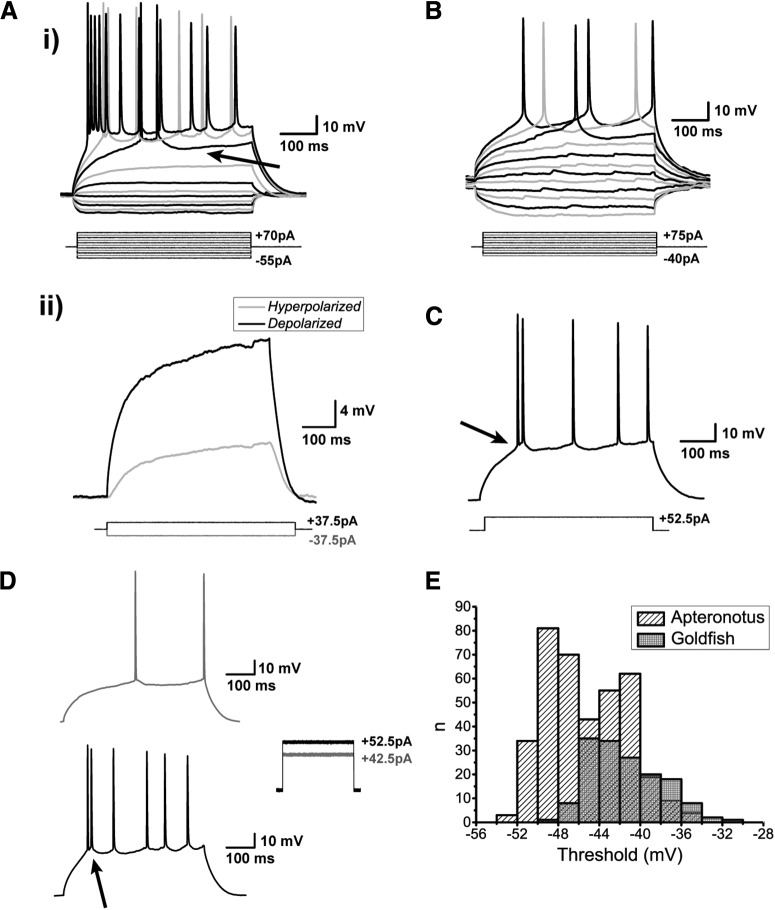
Spiking characteristics of DL neurons. ***Ai***, Example of an *Apteronotus* DL quiet neuron response to the injection of ±500-ms current-steps with varying amplitudes as shown below the response traces. The latency to the first evoked spike clearly decreases with increasing current intensities. However, even at elevated current injections (+70 pA), these cells cannot be driven to a high firing rate (maximum in this case was 22 Hz). This appears to be due, at least in part, to the prominent AHPs that follow the spikes (arrow). There is a large difference between the membrane potential responses to depolarizing versus hyperpolarizing current steps, much stronger responses are seen to positive current pulses. ***Aii***, We illustrate this asymmetry by superimposing the absolute responses to equal intensity injections of a hyperpolarizing and subthreshold depolarizing current steps; the response to the hyperpolarizing step is inverted for a clear comparison. DL neuron recordings in goldfish also yielded a similar asymmetry and spiking patterns (data not shown; but see panel ***B***). ***B***, Example of a goldfish dorsal DL (DLd) neuron response to a standard 500-ms current step injection; the region chosen for these recordings receive inputs from PG similar to the DL neurons in *Apteronotus.* The responses of these cells were very similar to those of *Apteronotus* DL neurons. ***C***, Example recording of a DL neuron in response to a single current step injection. The arrow highlights the location of the threshold for these neurons (panel ***E***). ***Di***, A single spike is evoked for currents near spike threshold. ***Dii***, After current injections induce depolarizations exceeding the spike threshold, DL neurons emit a short doublet or triplet burst of spikes at a shorter latency (arrow, *Apteronotus* recording; similar behavior was seen in goldfish DL neurons). Note that spike amplitude drops slightly but progressively in the ***C***, ***D*** traces. ***E***, Histogram of the average threshold of the first current-evoked spike in DL neurons. The spike threshold, which was found using the first derivative of the membrane potential, was ∼–45 mV in *Apteronotus* and ∼–42 mV in goldfish. The total number of spikes across all cells used for these estimates was *n* = 380 in *Apteronotus* and *n* = 154 in goldfish.

As in the work of Platkiewicz and Brette (2011), the membrane time constant, *τ* = *C*/*g_L_* = 5 ms, was introduced for our simulations. Given that the specific membrane capacitance is ∼ 0.9 µF/cm^2^ for practically all neuron types (Gentet et al., 2000), the leak conductance is constrained to be *g_L_* = 0.18 mS/cm^2^ and the input current, *I* = 3.8 nA, is scaled by the associated membrane resistance (5.56 MΩ). The leak current reversal potential was set to *E_L_* = –55 (Platkiewicz and Brette, 2011). When the membrane voltage reaches *θ* at time *t*, a spike is generated and *V*(*t* ^+^) is reset to the RMP, *V_r_* = –70 mV. The average threshold for the first spike in DL neurons was –42.96 ± 0.5 mV (*N* = 42 spikes). To obtain an approximate match between *V_T_* and this value, we kept the sodium activation slope, *k_a_* = 4 mV, and reversal potential, *V_a_* = –38.6 mV, at the empirically justified values used by Platkiewicz and Brette (2011). We then set the sodium conductance to *g_Na_* = 0.036 mS/cm^2^ to achieve a value of *g_Na_*/*g_L_* = 0.2, near the range of Platkiewicz and Brette (2011). We assume this slightly lower value in our model is a reflection of low sodium channel density. Consistent with this assumption, DL neuron axons are very thin and possibly unmyelinated (Giassi et al., 2012c) suggesting that they have a low sodium channel density, which may partly explains the high DL neuron threshold. The sodium channel reversal potential was kept at a standard *E_Na_* = 50 mV. When substituted into Equation 3, the above parameter set yielded an initial threshold of *V_T_* = –44.6 mV (see below in the Dynamic AHP and spike threshold section) and gave particularly close agreement with the *Apteronotus* data (–45.3 ± 0.2 mV; Fig. 4*E*).

Drawing on the Hodgkin–Huxley formalism, the inactivation variables, *h_f_* and *h_s_*, evolve according to Equations 4, 5, where *h*_∞_ is a Boltzmann equation with inactivation parameters *V_i_* = –63 mV and *k_i_* = 6 mV [6]:

(4) $$\tau_{f}\frac{dh_{f}}{dt}=h_{\infty }\left(V\right)-h_{f},$$

(5) $$\tau_{f}\frac{dh_{s}}{dt}=h_{\infty }\left(V\right)-h_{s},$$

(6) $$h_{\infty }\left(V\right)={\left(1+e^{\frac{V-V_{i}}{k_{i}}}\right)}^{-1}.$$

The parameters *τ_f_* (fast inactivation timescale) and *τ_s_*(slow inactivation timescale) are of particular interest to the model and to our results. To determine *τ_f_*, the average time between a short burst of two DL spikes (doublet) was measured at the beginning of the recorded voltage trace, yielding 15.38 ± 0.6 ms (*N* = 144 doublets). Selecting *τ_f_* = 15 ms, we note that the model generates spikes at a frequency of 64.7 ± 7.8 Hz, consistent with the data mean. We assumed that a slow timescale of inactivation would lead to an increase of spike threshold with a correspondingly long timescale for recovery (see Discussion). To select *τ_s_*, we therefore noted that the threshold for DL cell spiking remains significantly increased for at least 300 ms when stimulated; therefore, *τ_s_* is likely on the order of 10^2^ ms. A more direct estimate gave a mean decay time constant (τ_exp_) of ∼640 ms for the slow recovery (see ramp protocol results below). Therefore, we selected *τ_s_* = 500 ms, which is a conservative value, given slow inactivation is typically >1 s and longer timescales would only further strengthen our hypotheses (Itskov et al., 2011).

Note that we omitted Ca^2+^ currents and the resulting SK channel mediated AHP since its duration is less than the typical ISI of DL neurons. When simulating the model, subthreshold Gaussian noise, *N*(0,1), was added to Equation 1 and scaled by a factor *σ* = 0.5. The stochastic forward Euler method was used as the numerical solver.

### Code accessibility

The MATLAB code used in this paper is available as Extended Data 1 and at the University of Ottawa’s Institutional repository with the corresponding doi number: 10.20381/ruor39306. A Windows 10 computer was used to simulate the results from the iEIF model.

10.1523/ENEURO.0108-19.2019.ed1 Extended Data 1 MATLAB code for iEIF model. iEIF.zip contains the following files: iEIF.m is the main model file; h_inf.m is the Boltzman equation file (this equation is also present in iEIF.m); vline.m is a function file used to draw lines on the subplot figures. Download Extended Data 1, ZIP file.

All panel figures were initially compiled in OriginPro 9.0 (OriginLabs, RRID: SCR_015636) and the final figures were assembled in Adobe Illustrator CS6 (Adobe Systems, RRID: SCR_010279).

## Results

We performed whole-cell patch recordings from *Apteronotus* DL neurons in acute slices from the rostral- to mid-telencephalon (Fig. 1*A*). Cells within DL, imaged under infrared illumination with DIC optics, had a shape and size consistent with those identified in Nissl-stained sections (Fig. 1*B*,*C*). Although we cannot differentiate between excitatory and inhibitory cells, we assume that the neurons whose biophysical properties we characterize are almost certainly those of excitatory (glutamatergic) DL neurons since they vastly predominate over the rare inhibitory (GABAergic) cells (Giassi et al., 2012c). We also recorded neurons from the dorsal portion of *C. auratus* (goldfish) DL, while avoiding the ventral DL as it receives olfactory bulb input (Northcutt, 2006). The physiology of neurons recorded in the goldfish DL was not distinguishable from those of *Apteronotus* (see below).

### Noisy versus quiet cells

After attaining the whole-cell patch configuration, we first examined the RMP (no holding current), and observed two distinct electrophysiological profiles. The majority of the DL cells (29/35 cells in *Apteronotus* and 7/11 cells in goldfish) were quiet, that is, they had minimal spontaneous membrane fluctuations, as shown by the example recording traces from three different *Apteronotus* DL cells with different RMPs (Fig. 2*A*). A smaller number of DL neurons were noisy, showing considerable spontaneous membrane fluctuations over approximately the same range of RMPs as the quiet cells (Fig. 2*B*). A histogram estimating the distribution of RMP variance (Fig. 2*C*) suggests that, in both *Apteronotus* and goldfish DL, there were distinct populations of quiet (variance <0.5 mV^2^) and noisy cells (variance >0.5 mV^2^).

### Noisy cells

The noisy electrophysiological feature has previously been observed in pyramidal cells in the *Apteronotus* hindbrain ELL and has been attributed to the stochastic opening of voltage-gated ion channels, an effect which becomes stronger as the membrane potential increases toward threshold (Marcoux et al., 2016). We therefore wondered whether noisy DL cells shared these features. DL neurons displayed an *in vitro* RMPs that were relatively more hyperpolarized (*Apteronotus*: –70 to –84 mV; goldfish: –66 to –78 mV; Fig 2*D*), compared to the ELL pyramidal cells (–67.8 ± 5.7 mV, Berman and Maler, 1998) and neither subthreshold depolarizing, nor hyperpolarizing current steps altered the noise fluctuations of *Apteronotus* DL cells (*N* = 3 noisy cells; Fig. 2*E*). Additionally, we found that a more depolarized RMP of these noisy cells (*Apteronotus*, *N* = 6 cells; goldfish, *N* = 4 cells) was not associated with an increase in noise variance (Fig. 2*F*).

In some noisy cells, spontaneous membrane fluctuations could summate to cause a more sustained depolarization (Fig. 3*A*). The summating fluctuations were usually between 10 and 20 mV in amplitude and often induced spontaneous action potentials as the membrane potential crossed the spike threshold. The duration of these spontaneous events was estimated to be 425.5 ± 42.4 ms (*N* = 4 cells), and could reach as long as 800 ms in instances where spontaneous bursting occurred (Fig. 3*B*). We hypothesize that these events are caused by the summation of multiple postsynaptic potentials, as highlighted by the arrows in Figure 3*C*.

The intrinsic membrane noise of ELL pyramidal cells in the *Apteronotus* was shown to be unaffected by AMPA (CNQX) and NMDA (APV) receptor antagonists (Marcoux et al., 2016); this was expected given the lack of recurrent connections in ELL (Maler, 1979; Maler et al., 1981). In contrast, the application of kynurenic acid (10 mM), a broad spectrum AMPA/NMDA-R antagonist, completely blocked the membrane potential fluctuations of DL cells (*Apteronotus*: *N* = 3; goldfish: *N* = 2 cells; Fig. 3*D*); the average variance of the membrane potential decreased from 2.8 ± 0.9 to 0.10 ± 0.04 mV^2^ (paired *t* test; *p* = 0.0383, row a, Table 3; Fig. 3*E*) while having a negligible effect on the average RMP (paired *t* test; *p* = 0.7372, row b, Table 3; Fig. 3*F*).

Based on these observations, we suggest that the DL cell membrane noise, are not generated by intrinsic conductances, but are instead primarily due to synaptic bombardment from neighboring cells within the DL recurrent network (Trinh et al., 2016). In our slice preparation, DL is disconnected from all extrinsic input (Giassi et al., 2012a, b). As such, the synaptic noise we observed in a subset of DL neurons provides evidence that the activity of the DL recurrent network alone can drive weak spiking activity. We do not currently know why only some neurons show pronounced membrane potential fluctuations.

### Quiet cells

#### RMP, spike threshold, and spike discharge patterns

The RMPs of quiet *Apteronotus* DL cells were approximately Gaussian distributed with a mean of –76.7 ± 0.3 mV (*N* = 29 cells; Fig. 2*D*), similar to that of goldfish (–74.4 ± 0.7 mV, *N* = 7 cells). Using the hyperpolarized responses to negative current steps in the Apteronotus, we calculated an average membrane time constant of 10.28 ± 0.24 ms for these neurons.

We next injected positive current steps to generate spiking. An example recording is shown in Figure 4*Ai*, illustrating a typical DL neuron response in *Apteronotus*. The same response and spiking pattern was found in all cells regardless of their location within the *Apteronotus* DL region and was also observed in the goldfish DL (Fig. 4*B*). DL neurons exhibited very pronounced rectification: the membrane potential deflection in response to depolarizing current injections was far stronger than for hyperpolarizing currents of the same magnitude (Fig. 4*Aii*). This asymmetry is quantified below. In addition, we never observed any “sags” in the response of DL neurons to hyperpolarizing current injections, suggesting that they do not express hyperpolarization-activated cation channels (I_h_).

**Figure 5.**
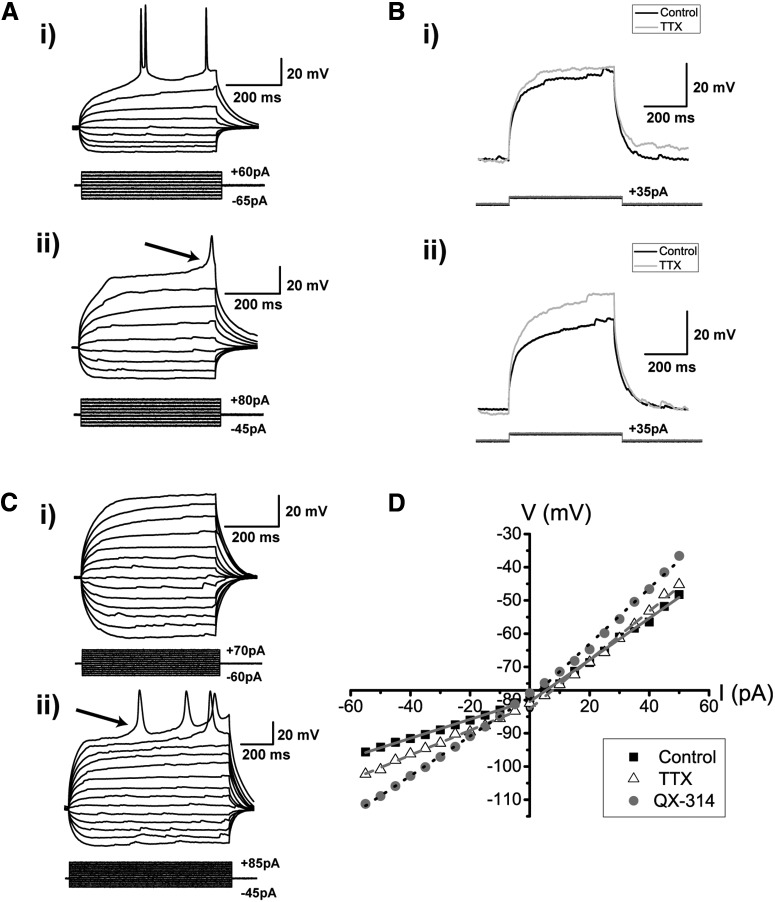
Pharmacological block of sodium and other channels in DL neurons. ***Ai***, This panel illustrates a goldfish DL neuron’s membrane potential response to 500-ms current step injections. For +60 pA, large spikes are evoked at a –38.4-mV threshold; in this example, the first spike has a height of 47.5 mV from the threshold and has a half-width of 3.1 ms. ***Aii***, The bottom panel shows the responses after bath application of 20 µM TTX, which completely eliminates the large fast spikes. Delayed, broad spikes (amplitude: 22.7 mV from the threshold, half width: 10.5 ms) are now evoked at elevated current levels (+80 pA) with a spike threshold of –21.0 mV. The arrow indicates the approximate location of the threshold for the broad TTX-insensitive spike. ***Bi***, The response of a DL neuron to a current step at the subthreshold membrane potential before (black) and after (gray) application of TTX. After TTX treatment, the membrane potential did not dramatically change compared to control, and in some cases, (***Bii***) the subthreshold membrane potential was even more depolarized than in the control condition. ***Ci***, Response of an *Apteronotus* DL neuron to current injection steps following QX-314 application via the recording pipette. Fast Na^+^ spikes are eliminated by this treatment, even with strong current injections (+70 pA, 500 ms) that would always evoke spiking in control neurons. ***Cii***, Stronger current injection (+85 pA, 1000 ms), evoked delayed broad spikes (amplitude from the threshold = 36.9 mV, half-width; 25.9 ms) with a higher threshold (–8.3 mV) compared to the TTX-insensitive spikes illustrated in panel ***Ai***. Stronger current injections (+85 pA, 1000 ms) evoked several putative Ca^2+^ spikes with a shorter latency to the first spike. The arrow highlights the approximate location of the threshold of the broad Ca^2+^ spike. ***D***, Average I-V relationship obtained from subthreshold *Apteronotus* and goldfish DL recordings without the application of any pharmacological blockers (black squares), after the application of 20 µM TTX (white triangles), and with the inclusion of QX-314 within the patch pipette solution (gray circles). Both the curves for control and TTX are piecewise linear with the slope being markedly smaller for hyperpolarizing (control; 0.28 ± 0.02 mV/pA, TTX; 0.32 ± 0.04 mV/pA) compared to depolarizing steps (control; 0.69 ± 0.03 mV/pA, TTX; 0.74 ± 0.03 mV/pA). In contrast, the addition of QX-314 has linearized the I-V curve (hyperpolarizing slope = 0.60 ± 0.11 mV/pA, depolarizing slope = 0.86 ± 0.12 mV/pA) with its main effect on the response to hyperpolarizing current injections (Table 1). Extended information illustrating the expression of GIRK channels in the *Apteronotid* fish’s brain is available in Extended Data Figure 5-1.

DL neurons discharge very few action potentials (Fig. 4*A*,*B*) and the average injected current necessary to reach spike threshold (rheobase) was 38.17 ± 2.52 pA (*N* = 15 cells). Strong current injections (70 pA) only resulted in average firing rates of 15.3 ± 2.4 Hz (*N* = 15 cells). We defined the spike threshold as the voltage corresponding to a pre-determined fraction of the maximal peak of the first derivative of the membrane potential response to current steps (Azouz and Gray, 2000; see Materials and Methods). Strong current injection in *Apteronotus* DL neurons typically results in an initial high-frequency burst of two or three spikes, followed by an irregular series of spikes separated by AHPs of varying amplitude and duration (Fig. 4*C*,*D*); the same pattern was also observed in the DL of goldfish (Figs. 4*B*, 5*A*). In *Apteronotus*, the threshold for the first spike is distributed with a mean of –45.3 ± 0.2 mV (*N* = 22 cells) and has a high degree of overlap with the observed spike threshold for goldfish DL cells (mean: –41.5 ± 0.3 mV, *N* = 14 cells; Fig. 4*E*). We measured the mean spike peak amplitude from both the membrane potential at spike threshold (*Apteronotus*: 66.2 ± 1.0 mV, *N* = 22 cells; goldfish: 50.8 ± 1.0 mV, *N* = 14 cells) and from the RMP (*Apteronotus*, 95.9 ± 0.5 mV; goldfish, 90.6 ± 0.5 mV). Lastly, we also measured the spike half-width at half-maximum (*Apteronotus*: 2.3 ± 0.1 ms; goldfish: 3.7 ± 0.3 ms).

10.1523/ENEURO.0108-19.2019.f5-1 Extended Data Figure 5-1 GIRK channel mRNA expression obtained from RT-PCR in the *Apteronotus* brain using pan-PCR primer pairs in conserved regions. GIRK channels are ubiquitously expressed albeit at variable levels. In particular they are expressed in DL. SP, subpallium; TT, tectum/torus; Cer, cerebellum; HB, hindbrain; ch, chicken (negative control); M, molecular marker. Download Figure 5-1, TIF file.

In summary, the core biophysical properties of DL cells receiving PG input in *Apteronotus* and goldfish (dorsal DL, non-olfactory; Northcutt, 2006; Yamamoto and Ito, 2008) were similar, DL neurons have a hyperpolarized RMP and a high spike threshold and spike only sparsely in response to even strong current injection.

### Asymmetric input resistance

A striking property of *Apteronotus* and goldfish DL cells is an asymmetry in their response to hyperpolarizing versus depolarizing current steps (Fig. 5*Ai*). In ELL pyramidal cells, an equivalent, though far smaller asymmetry is caused by a persistent Na^+^ channel (Turner et al., 1994) that amplifies excitatory synaptic input (Berman et al., 2001). We tested this possibility by blocking the sodium channels of DL neurons with a local application of 20 µM TTX (control: *N* = 18 cells, TTX: *N* = 6 cells). As expected, spike discharge at the previous threshold (∼–45 mV) was completely blocked by TTX (Fig. 5*Aii*); the small high-threshold spikes evoked with much stronger current injections will be discussed below (Fig. 5*Aii*,*Cii*). On closer inspection of the neurons’ response to positive current injections, we found that application of TTX did not dramatically change their depolarizing ramp response to peri-threshold current injection (Fig. 5*Bi*) and, in some cases, would even slightly increase the neuron’s response to positive current injections (Fig. 5*Bii*). These data indicate that low threshold persistent sodium channels are likely not (or only weakly) expressed in DL neurons.

We next plotted the average I-V curves for negative and positive (subthreshold) current injection (*Apteronotus* and goldfish; Fig. 5*D*). The stronger response to positive versus negative current injection can be clearly seen in the rectification of the I-V curve for the control condition. These curves can be used to compute separate input resistances for positive and negative current injections. Typically, the response to hyperpolarizing current injection is assumed to reflect the passive properties of a neuron and is reported as its input resistance (e.g., ELL pyramidal cells; Mathieson and Maler, 1988; Berman et al., 1997). In DL cells, the input resistance for depolarizing current injection is approximately double that for hyperpolarizing current injection when compared under both control and TTX conditions (Table 1; paired *t* test; control; *p* = 3.3 × 10^−12^, row c, Table 3, TTX; *p* = 9.9 × 10^−6^, row d, Table 3). The addition of TTX had no significant effect on the hyperpolarizing slope (one-way ANOVA; *p* = 0.32, row f, Table 3), nor did it have any significant effect on the input resistance for the depolarizing slope (Table 1; one-way ANOVA; *p* = 0.42, row h, Table 3; Fig. 5*D*). Thus, it appears that there is no contribution of persistent Na^+^ channels to the RMP of DL neurons, in accordance with the small effects of TTX observed in Figure 5*B*.

**Table 1.** I-V slope measurements obtained from the depolarizing and hyperpolarizing responses of DL neurons in both teleost species for the TTX and QX-314 experiments

Conditions	Depolarizing slope (GΩ)	Hyperpolarizing slope (GΩ)
Control (*N* = 18 cells)	0.69 ± 0.03	0.28 ± 0.02
TTX (*N* = 6 cells)	0.74 ± 0.03	0.32 ± 0.04
QX-314 (*N* = 6 cells)	0.86 ± 0.12	0.60 ± 0.11

To further investigate the basis of the observed asymmetrical response to current injection, we have also recorded DL neurons using an intracellular solution containing 5 mM QX-314, a blocker of Na^+^ channels, as well as some K^+^ and Ca^2+^ channels (Talbot and Sayer, 1996; control, *N* = 18 cells; QX-314, *N* = 6 cells; Fig. 5*C*,*D*). QX-314 has previously been used to block all Na^+^ channels in *Apteronotus* ELL pyramidal cells (Berman et al., 2001). The I-V graph constructed from the QX-314 experiments showed a higher depolarizing versus hyperpolarizing input resistance (paired *t* test; *p* = 2.3 × 10^−4^, row e, Table 3), similar to control and TTX conditions (above). There was a small increase in input resistance for the depolarizing current injection that failed to reach significance (Table 1; one-way ANOVA, *p* = 0.07, row i, Table 3; Fig. 5*D*). In contrast, there was a large and highly significant increase of input resistance in the responses to hyperpolarizing current injections, it more than doubled over control values (Table 1; one-way ANOVA, *p* = 5.9 × 10^−5^, row g, Table 3). Since we only expect K^+^ permeating channels to be open at such hyperpolarized membrane potential, we attribute this effect to the “non-specific” actions of QX-314 (Perkins and Wong, 1995; Slesinger, 2001). The results of the TTX and QX-314 experiments lead to two hypotheses: first, the subthreshold response of DL cells to depolarizing input is mainly due to their passive membrane properties. Second, the RMP of hyperpolarized DL cells is likely due to a strong rectifying K^+^ conductance that is blocked by QX-314 and typically prevents the cell from deviating from the reversal potential of K^+^ ions (Fig. 5). Given that GIRK channels are ubiquitous in the mammalian cortex (Lüscher and Slesinger, 2010; Luján and Aguado, 2015) and can be blocked by QX-314 (Zhou et al., 2001), we suspected them to also be present in the teleost pallium. To confirm the presence of GIRKs in DL, we used a RT-PCR approach to show the expression of GIRK channels in different brain regions in the *Apteronotid* fish (DL, subpallium, tectum/torus, cerebellum, ELL and hindbrain) using a primer pair hybridizing in conserved segments of all GIRK paralogs. Unsurprisingly, pan-GIRK amplicons were found in all brain regions, but were not present in the control (Extended Data Fig. 5-1), suggesting that GIRK channels are ubiquitously expressed in the *Apteronotus* brain.

### Voltage-dependent calcium conductance

In the presence of TTX, strong current injections (>80 pA) were able to evoke a broad (half-width: 33.0 ± 3.1 ms) spike with a very high threshold (mean threshold: –21.2 ± 0.5 mV, *N* = 4 of 6 cells; Fig. 5*Aii*). Spike amplitude was 18.6 ± 0.7 mV from the threshold potential and 79.1 ± 0.9 mV from the RMP. Similar to the TTX results, QX-314 treated cells did not produce any action potentials at the threshold for control cells (Fig. 5*Ci*), but did produce broad spikes at much higher stimulus intensities (spike half-width, 21.1 ± 1.1 ms; height = 31.3 ± 0.7 mV from threshold and 100.6 ± 0.9 mV from RMP, *N* = 4 of 6 cells; Fig. 5*Cii*). The average threshold for these broad spikes was found to be at –6.8 ± 1.3 mV, which is also consistent with the range of voltages that has been reported for the activation of HVA Ca^2+^ channels (Tsien et al., 1988). Therefore, we hypothesize that DL neurons express HVA Ca^2+^ channels that will likely be activated by Na^+^-mediated action potentials.

### AHPs

**Figure 6.**
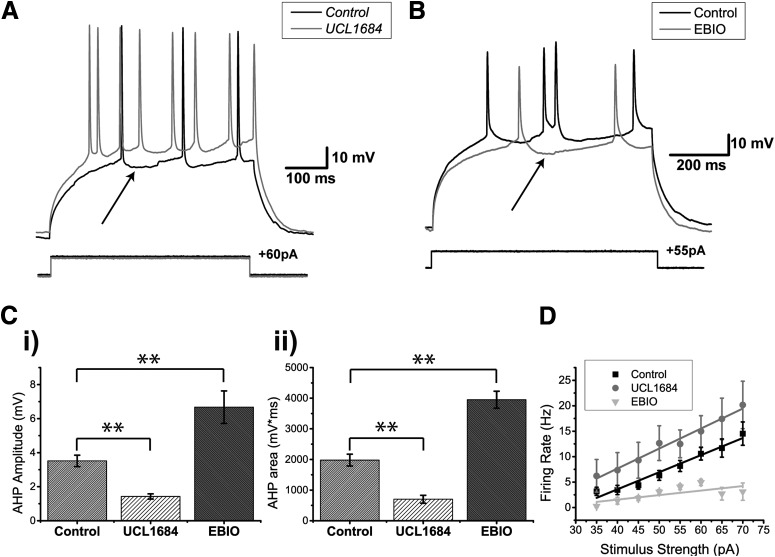
SK-mediated potassium channels contribute to the AHP of DL neurons. ***A***, *Apteronotus* DL neuron response to 500-ms step current injection before (black trace) and after the bath application of 30 µM UCL1684 (gray trace). The black arrow shows the minimum membrane potential between two spikes and is used to estimate the amplitude of the AHP by comparison with the membrane potential immediately preceding the first action potential. The prominent AHPs seen in the control condition are reduced by this treatment and the spike rate has also increased (from three to eight spikes). ***B***, DL neuron response to the injection of 750-ms current steps before (black trace) and after bath application of 1 mM EBIO in the goldfish (gray trace; a longer pulse was needed to increase the likelihood of evoking more than one spike). The amplitude of the AHP (arrow) was increased by this treatment and the spike rate has been reduced (from 4 to 2 Hz). ***C***, Average amplitude (***i***) and average area under the membrane potential (***ii***) of the AHP following the first spike of DL neurons in response to current steps (control, *N* = 12 cells; UCL1684, *N* = 7 cells; EBIO, *N* = 6 cells). Both the amplitude and the area under the AHP are significantly diminished after the application of UCL1684, while a strong increase was observed after the application of EBIO. ***D***, Average firing rate plotted as a function of the amount of current injected for the control condition (black trace), the UCL1684 condition (gray trace), and the EBIO condition (light gray trace) in both *Apteronotus* and goldfish (control, *N* = 28 cells; UCL1684, *N* = 7 cells; EBIO, *N* = 6 cells). The firing rate increases for all current injections after UCL1684 application, while the firing rate decreases after the EBIO application. **p < 0.01.

DL neurons exhibit a strong AHP (Figs. 4–6). Previously, it was shown that DL cells express both SK1 and SK2 channels (Ellis et al., 2008) and that UCL1684 is highly effective at blocking such channels (Harvey-Girard and Maler, 2013). We therefore bath-applied 30 µM UCL1684, resulting in a significantly diminished AHP compared to the control conditions (Fig. 6*A*). To quantify this AHP reduction, we measured the AHP amplitude (Fig. 6*Ci*) and the area under the AHP (Fig. 6*Cii*) following the first single spike obtained in response to current injection. The addition of UCL1684 reduced the average amplitude of the first AHP to half its control value (control: 3.5 ± 0.3 mV, *N* = 13 cells; UCL1684: 1.4 ± 0.2 mV; *N* = 7 cells; two-sample *t* test; *p* = 0.0003, row j, Table 3; Fig. 6*Ci*). A similar reduction was also observed when comparing the area under AHPs: from 1980.3 ± 192.6 to 701.4 ± 128.4 mV/ms (two-sample *t* test; *p* = 0.0002, row l, Table 3; Fig. 6*Cii*). In contrast, after the addition of the SK channel agonist EBIO (1 mM; Ellis et al., 2007), current injection evoked very few spikes; thus current steps were increased to 750 and 1000 ms. As expected, the average AHP amplitude increased from 3.5 ± 0.3 mV to 6.7 ± 1.0 mV (control, *N* = 13 cells; EBIO, *N* = 6 cells; two-sample *t* test; *p* = 0.001, row k, Table 3), while the area under the curve also increased from 1980.3 ± 192.6 to 3952.2 ± 277.5 mV/ms (two-sample *t* test, *p* = 0.00002, row m, Table 3).

Blocking SK channels also increased the current-evoked firing rate compared to the control condition (control, *N* = 28 cells; UCL1684, *N* = 7 cells; two-way ANOVA; *p* = 0.0013, row n, Table 3; Fig. 6*D*), while EBIO reduced the evoked firing rate since the cell required a longer time to reach spike threshold after the first spike (*N* = 6 cells; two-way ANOVA; *p* = 0.000092, row o, Table 3; Fig. 6*B*,*D*). We conclude that the SK1/2 channels of DL neurons act as negative feedback on the cell’s responsiveness to excitatory input.

**Figure 7.**
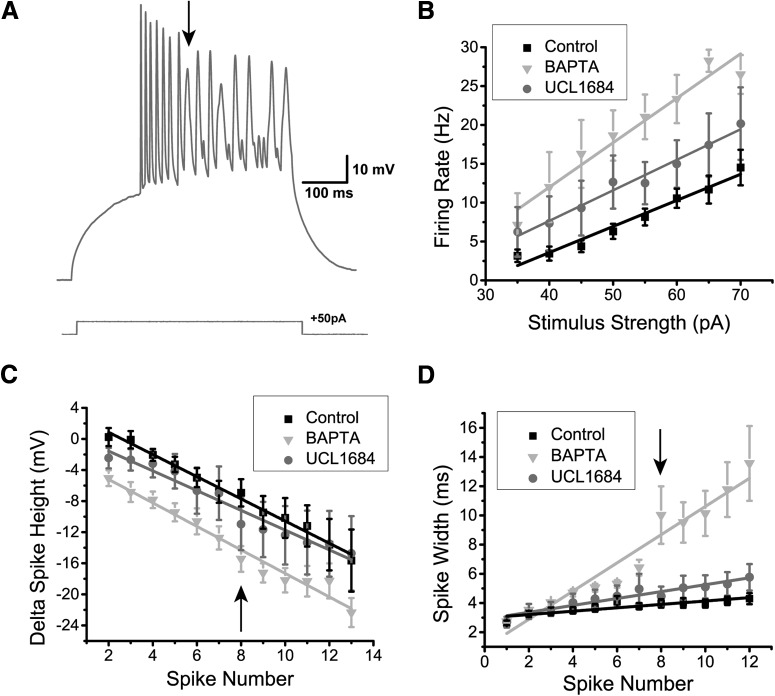
The effect of intracellular Ca^2+^ chelation on DL neuron responses to depolarization. ***A***, Example recording trace (*Apteronotus*) with 10 mM BAPTA added to the internal solution of the patch pipette. The AHP appears to be completely eliminated which promotes higher frequency spiking; note that successive spike heights drop continuously for the first seven spikes. By the 8th spike, very prominent spike broadening begins and the spike height drops to an even greater degree compared to the UCL1684 application in Figure 6*A*. ***B***, The average firing rate was plotted as a function of the amount of current injected for the control (black trace, *N* = 28 cells), UCL1684 (gray trace, *N* = 7 cells), and BAPTA conditions (light gray trace, *N* = 7 cells). The addition of intracellular BAPTA promotes an even stronger increase in firing rate compared to the addition of the SK channel blocker UCL1684. ***C***, The average difference in spike height between the *n*th spike and the first spike was plotted as function of successive spikes obtained after a 500-ms current step injection for all three conditions mentioned in ***B***. The addition of UCL1684 did not strongly affect the spike height, unlike the addition of BAPTA, which reduced the spike height across successive spikes following a step current injection. The arrow highlights the 8th spike, which marks the beginning of the non-linearity in the BAPTA condition. ***D***, The average spike width was plotted as a function of successive spikes, similar to panel ***C***. UCL1684 application has only a minimal effect on spike width. The presence of intracellular BAPTA increased the spike width across successive spikes during a step current injection when compared to the other conditions. The arrow indicating the 8th spike marks a strong change in spike width, as denoted by the arrow in ***A***.

Finally, we wanted to confirm whether SK channel activation in DL neurons could be blocked by preventing Ca^2+^ activation of the channel. We recorded DL neurons in *Apteronotus* using an intracellular solution containing 10 mM BAPTA, a Ca^2+^ chelator (*N* = 7 cells; Fig. 7*A*). In all cases, the AHP was completely abolished, unlike the partial AHP block obtained with UCL1684. This suggests that another unidentified Ca^2+^-activated K^+^ channel may also be contributing to the AHP. Further work will be required to investigate this possibility. The firing rate also dramatically increased compared to the control condition (control, *N* = 28; UCL1684, *N* = 7; BAPTA, *N* = 7 cells; two-way ANOVA; *p* = 1.5 × 10^−15^, row p, Table 3; Fig. 7*B*) and compared to UCL1684 treatment (two-way ANOVA; *p* = 0.00063, row q, Table 3). Furthermore, this BAPTA-induced increase in firing rate was also accompanied by a significant reduction in spike height compared to both control (two-way ANOVA; *p* = 2.1 × 10^−12^, row r, Table 3; Fig. 7*C*) and UCL1684 conditions (two-way ANOVA; *p* = 1.6 × 10^−6^, row s, Table 3). In contrast, the difference in spike height between the UCL1684 and control did not yield a significant difference (two-way ANOVA; *p* = 0.14, row t, Table 3). We hypothesize that Na^+^ channel inactivation may be causing this reduction (see below in the Dynamic AHP and spike threshold section). Another distinctive feature of the DL neuron’s spiking response during the BAPTA application was the increase in spike width occurring along successive spikes and typically becoming most prominent by the 8^th^ spike (Fig. 7*A*,*D*). In the control and UCL1684 conditions, there was a slight increase in spike width, however, in the BAPTA condition, the spike width increased dramatically with successive spikes (Fig. 7*A*,*D*) compared to control (two-way ANOVA; *p* = 1.3 × 10^−31^, row u, Table 3) and UCL1684 conditions (two-way ANOVA; *p* = 3.7 × 10^−10^, row v, Table 3). In contrast, the difference between the control and BAPTA conditions was not significant up until the third spike (two-way ANOVA; *p* = 0.24, row w, Table 3), suggesting that the spike width increase is caused by a cumulative process. Calcium channels typically inactivate via a Ca^2+^-dependent mechanism (Simms and Zamponi, 2014), leading us to hypothesize that this dramatic change in spike width may be caused by a decrease in Ca^+^-dependent inactivation of the Ca^2+^ channel leading to an increase of its open time.

### Dynamic AHP and spike threshold

**Figure 8.**
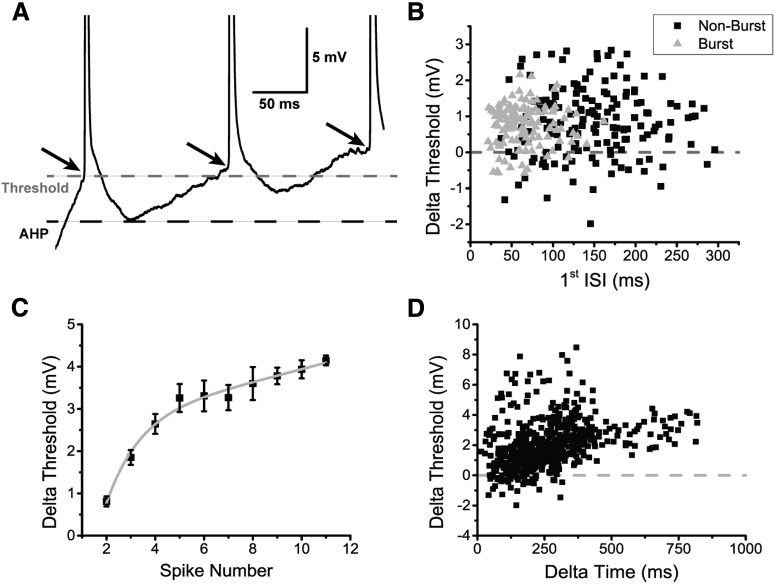
DL neuron spiking causes a decrease in AHP amplitude and an increase in spike threshold. ***A***, A magnified view of the first three spikes in an example trace of a DL neuron’s response to a +60-pA current injection where the black dashed line is placed to coincide with the minimum of the first spike’s AHP and the gray dashed line is placed to coincide with the first spike’s threshold. The black arrows highlight the progressive increase in spike threshold following consecutives spikes. ***B***, The increase in spike threshold between the second and first spikes was plotted in the same manner as a function of the first ISI. Individual black squares represent a pair of spikes that were taken from a trace which did not contain an initial burst (total of 160 non-burst pairs), while individual gray triangles represent a pair of spikes that were taken from a trace displaying an initial burst of spikes as in Figure 4*C* (total of 117 burst spike pairs). The majority of the spike thresholds increased (>300 ms) with no evident recovery. ***C***, The difference in average spike threshold between the *n*th spike and the first spike is plotted as a function of the spike number. The subsequent curve was fit with a double exponential equation (y = 2.72*e* ^0.04x^ –9.0*e* ^− 0.72x^; *R* ^2^ = 0.987). ***D***, The increase in spike threshold between the *n*th spike and the first spike is plotted as a function of the time interval between them. Each black square represents a spike pair (total of 573 spike pairs). Overall, the increase in threshold appears to be larger following longer timer intervals. Extended information related to the modulation of the AHP after prolonged spiking is available in Extended Data Figure 8-1.

Although the presence of the AHP greatly reduces the firing rate, we also observed that after successive spikes, the AHP itself decreased (Extended Data Fig. 8-1*A*) and the spike threshold increased (Fig. 8*A*). To better quantify the AHP modulation, we measured the difference in AHP amplitude between the first two spikes of a current-evoked spike train that did not show an initial burst. We found that there was a significant reduction in AHP amplitude that was invariant to the time length of the AHP (*N* = 26 cells; one-sample *t* test, *p* = 4.45 × 10^−27^, row x, Table 3; Extended Data Fig. 8-1*B*, black squares). For recording traces that showed initial bursts, we examined the first spike pair following the burst and found a similar reduction in AHP (*N* = 20 cells; one sample *t* test, *p* = 1.26 × 10^−13^, row y, Table 3; Extended Data Fig. 8-1*B*, gray triangles). This reduction is presumably caused by Ca^2+^-induced inactivation of the HVA Ca^2+^ channels, which will decrease the total amount of Ca^2+^ available to the cell and limit the activation of SK channels.

10.1523/ENEURO.0108-19.2019.f8-1 Extended Data Figure 8-1 GIRK channel mRNA expression obtained from RT-PCR in the *Apteronotus* brain using pan-PCR primer pairs in conserved regions. GIRK channels are ubiquitously expressed albeit at variable levels. In particular they are expressed in DL. SP, subpallium; TT, tectum/torus; Cer, cerebellum; HB, hindbrain; ch, chicken (negative control); M, molecular marker. Download Figure 8-1, TIF file.

Even with the spiking-induced reduction of the AHP, DL neurons could not surpass a sustained firing rate of 30 Hz (Fig. 7*B*), which suggests the presence of an additional mechanism(s) that limits firing rate. In ELL pyramidal neurons, spike threshold fatigue has been shown to limit the firing rate whenever a burst occurs (Chacron et al., 2007). On closer inspection, we found a significant increase in spike threshold during long spike trains (Fig. 8*A*). This dynamic spike threshold was also found to be invariant to the ISI (up to ∼300 ms) for both non-burst traces (one-sample *t* test, *p* = 1.24 × 10^−22^, row z, Table 3; Fig. 8*B*, black squares) and for traces containing an initial burst (one sample *t* test, *p* = 8.30 × 10^−28^, row aa, Table 3; Fig. 8*B*). Next, we wanted to confirm whether the threshold fatigue that was observed in DL neurons may be caused by the history of past spikes, i.e., whether Na^+^ channel inactivation due to continuous spiking may influence the spike threshold. We examined the difference in threshold for all non-burst traces to see whether it varies throughout a spike train. The threshold increases continued to at least 10 spikes and could be fitted by a double exponential function (equation: y = 2.72*e*^0.04x^ + –9.0*e* ^− 0.72x^; *R* ^2^ = 0.987; Fig. 8*C*); here we considered only the number of spikes and not the duration of the spike train. A similar analysis where the difference in threshold was compared to the time between spikes instead of the spike number also led to the same conclusion: this effect became more prominent after long periods of depolarization despite the variability in the number of intervening spikes (Fig. 8*D*). These results suggest that the increase in threshold is caused by an accumulation of slow Na^+^ channel inactivation.

**Figure 9.**
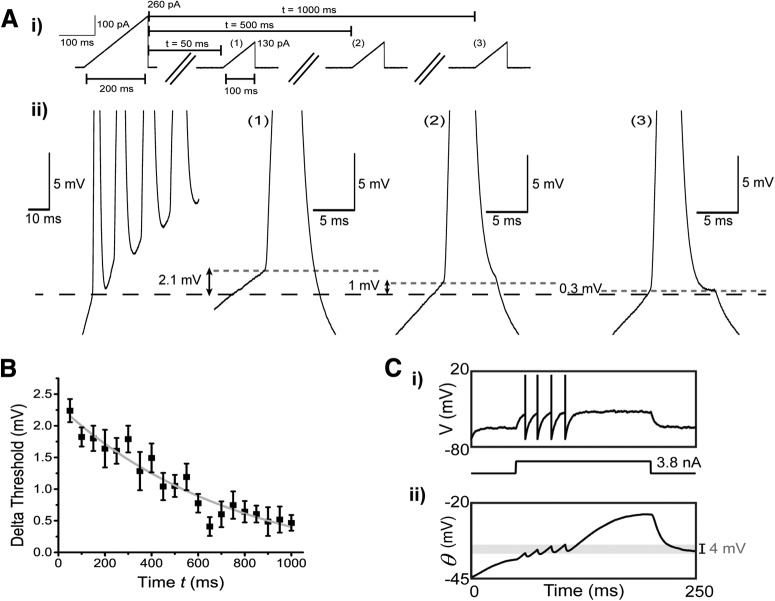
DL neuron spike threshold adaptation can last up to hundreds of milliseconds. ***Ai***, Two ramp current injections separated at various times *t* (in this example 50, 500, and 1000 ms) were used to measure the time constant of the spike threshold adaptation. Although both ramp current injections have the same slope (slope = 1.3 pA/ms), the objective of the first ramp current was to induce an accumulation of Na^+^ channel inactivation through the firing of multiple action potentials and therefore was stronger than the second ramp current which would only produce one action potential. ***Aii***, A magnified view of the example responses obtained after the first ramp current injection and after the second ramp current injections at times *t* = 50 ms, *t* = 500 ms, and *t* = 1000 ms, respectively. The black dash line is aligned to the first spike’s threshold obtained after the first ramp current injection while the gray dotted lines are aligned to the spike threshold obtained from the second ramp current injection for the various times *t* mentioned previously. ***B***, The average difference in spike threshold (for the first spike only) between the first and second ramp current injections were plotted as a function of the time *t* between each ramp injection. The resulting curve was fitted with an exponential equation (y = 2.38*e^−0.0017x^, *R* ^2^ = 0.917). ***C***, We used a simplified EIF with fast (*τ_f_* = 15 ms) and slow (*τ_s_* = 500 ms) Na^+^ channel inactivation in an attempt to connect the apparent Na^+^ channel inactivation (Fig. 7*A*) with the increase in spike threshold over multiple spikes (Fig. 8*C*,*D*). ***i***, When driven by a step current, the model produces a small number of spikes at frequencies consistent with the data; however, the neuron quickly ceases discharge despite continuous application of the strong positive current. ***ii***, This result can be easily understood in terms of the dynamic spike threshold (*θ*), which increases because of cumulative slow inactivation of the Na^+^ channel (*h_f_* and *h_s_* not shown). Note that the threshold changes by ∼4 mV (gray shading) over the course of a few spikes, in line with the upper bound for threshold increases seen between DL cell spikes (Fig. 8*C*). Our model parameter set gave an initial threshold of −44.6mV in close agreement with the *Aptoronotus* data (−45 mV, Fig. 4*E*).

In mammalian cortical cells, the recovery from inactivation of Na^+^ channels have both a fast component (millisecond timescale) and a slow component that can extend to much longer timescales (seconds to minutes; Fleidervish et al., 1996; Mickus et al., 1999; Ellerkmann et al., 2001). To quantify the duration of this spike threshold adaptation, we developed a protocol in which a long ramp current (evoking multiple spikes) was injected followed by a shorter ramp current (evoking one spike) at various inter-stimulus time intervals (Fig. 9*A*, upper panel). This protocol induced spike threshold fatigue during the first current injection, while the second current injection was used to test for time-dependent changes in spike threshold. We found that the increase in spike threshold between the first and second current injection was significantly higher at short compared to longer time intervals (Fig. 9*A*, bottom panel). Using these changes in threshold, we found that the recovery from this spike threshold fatigue had a highly variable time constant ranging from 300 to 900 ms with an average time constant τ_exp_ = 637.28 ± 85.9 ms (Fig. 9*B*). This suggest that the decrease in cell excitability caused by the dynamic threshold can operate on the timescale of hundreds of milliseconds.

To further investigate whether Na^+^ channel inactivation is responsible for the observed increase in threshold, we employed a model with minimal assumptions: the iEIF (Platkiewicz and Brette, 2011). This model includes a slow inactivation term, as well as the traditional fast inactivation term associated with Na^+^ channels; inactivation kinetics for both forms were derived from our data (see Materials and Methods). Since we were primarily interested in the effect of sodium inactivation on the spike threshold, the minimal model omits AHP dynamics and Ca^2+^ currents (see Materials and Methods). We found that the addition of slowly inactivating Na^+^ channels, as suggested by the effects of BAPTA (Fig. 7*A*), was itself sufficient to qualitatively reproduce the response of DL neurons to current injection and predict an increase in spike threshold that was similar to that observed in our whole-cell recordings (Fig. 9*C*). We therefore conclude that the accumulation of slow Na^+^ channel inactivation, caused by spike discharge and simple depolarization, may act as a source of negative feedback to reduce the cell’s firing rate via an increase in spike threshold.

## Discussion

The work presented here is, to our knowledge, the first study of the biophysical properties of teleost DL neurons. Our previous work mapped the local DL circuitry (Trinh et al., 2016), the organization of thalamic and other inputs to DL (Giassi et al., 2012b), and the telencephalic connectivity of DL (Giassi et al., 2012a; Elliott et al., 2017). In gymnotiform fish, we have previously shown that the electrosensory system contributes to spatial learning (Jun et al., 2016), and that the PG (thalamic) inputs to DL can encode responses to object motion (Wallach et al., 2018). Although the intrinsic and extrinsic properties of DL synaptic input have yet to be examined, we believe that the constraints imposed by DL circuitry, behavioral function plus recent theoretical analyses, are sufficient to generate testable hypotheses of the computations performed by DL during spatial learning. Below, we first summarize the main conclusions of our work and then discuss whether the biophysical properties of DL neurons and their connectivity are compatible with the critical role of DL in spatial learning and memory. In particular, we suggest that DL neurons possess the minimal requirements to be labeled as sparse coders. Next, we suggest that spike threshold adaptation is key to the extraction of spatial information in DL from the time stamped electrosensory input conveyed by PG (Wallach et al., 2018). Our hypothesis relies on a previous theoretical model of time coding cells (Itskov et al., 2008) that utilizes, as an essential ingredient, spike threshold adaptation with a long recovery time constant.

Our main results show that DL neurons express a combination of ion channels that have been reported for many other types of neurons. DL neurons have a hyperpolarized RMP. We hypothesize that this is due, at least in part, to GIRK channels. GIRKs can hyperpolarize mammalian CA1 hippocampal neurons by at least 8 mV under basal conditions (Lüscher and Slesinger, 2010) and have been shown to set the RMP of dorsal cochlear nucleus (DCN) neurons to a hyperpolarized level (Ceballos et al., 2016). DL neurons also have a high spike threshold and theoretical analyses suggest this may attributed to a low density of voltage-gated Na^+^ channels (Platkiewicz and Brette, 2010). Furthermore, our results also imply the presence of HVA Ca^2+^ channels, which activate a strong SK channel-mediated AHP that strongly reduces current-evoked spiking. We propose that the combination of a hyperpolarized RMP, the low input resistance at hyperpolarized potentials (Table 1), a high spike threshold and strong AHPs will greatly reduce DL cell excitability and therefore prevent incoming excitatory synaptic input from driving strong spiking responses.

An unusual and, we believe, critical feature of DL neurons is that they exhibit long-lasting spike threshold adaptation (i.e., threshold fatigue); our modeling suggests that this is due to Na^+^ channels exhibiting slow recovery from inactivation. In mammalian cortical neurons, the Na^+^ channel’s slow recovery from inactivation can last up to a few seconds and can regulate the neuron’s excitability; in particular, the slow inactivation of dendritic Na^+^ channels in CA1 neurons can attenuate back-propagating action potentials (Jung et al., 1997). In addition, the link between a sustained spike threshold increase and the slow inactivation of Na^+^ channels has previously been suggested for hippocampal CA1 pyramidal neurons (Henze and Buzsáki, 2001). This spike threshold adaptation mechanism was later used to model time cells using a recurrent network model (Itskov et al., 2011; see below). We note that the AHP and slowly recovering Na^+^ inactivation have very different effects on neuron excitability (Benda et al., 2010). The dynamical interaction of these biophysical mechanisms (not currently known) will likely be a critical determinant of the spiking response of DL neurons to their time varying synaptic input. Developing a high quality model of DL cells will be an essential next step in connecting the dynamics of the DL recurrent network (Trinh et al., 2016) to *in vivo* imaging/recording and behavioral studies on spatial learning in the dark (Jun et al., 2016).

### The biophysical properties of DL neurons suggest that they are sparse coders

The main properties that contribute to low DL neuron firing rates are the very depolarized spike threshold and hyperpolarized RMP (Table 2); these parameters are highly variable but typically lead to a large (∼32 mV) barrier that excitatory input must exceed to evoke spiking (Table 2). This contrasts sharply with the first order electrosensory pyramidal cells within the ELL. Their barrier from rest to spiking is a mere 4.9 mV (Table 2) and they can even respond to weak signals with discharge frequencies over 100 Hz. ELL pyramidal neurons also recover rapidly from spike induced increases in spike threshold, i.e., threshold fatigue (tens of milliseconds; Chacron et al., 2007). It is hypothesized that these properties are responsible for the ability of pyramidal cells to densely encode spatial and social electrosensory signals (Vonderschen and Chacron, 2011). The low barrier from RMP to spike threshold is also seen in primary auditory neurons and in layer 4 cells of the primary visual and somatosensory cortex (Table 2). Although no precise estimates are available, it appears likely that all these low-level sensory neurons encode sensory input much more densely than neurons in the hippocampus.

**Table 2.** Difference in spike threshold and resting membrane across multiple cell types

	“Hippocampus”		L4 sensory cortex		Primary sens. cells	
Values in mV	DL cells	DG granule cells^a^	Barrelfield^b^	Visualcortex^c^	ELL PyrON cells	DCN PyrCells^f^
Spike threshold	–45.3	–40.8	–45.1	∼–63.5	–62.9^d^	–48.1
RMP	–76.7	–74.7	–63.0	–72.0	–67.8^e^	–62.7
Threshold–RMP	31.4	33.9	17.9	8.5	4.9	14.6

a Kowalski et al. (2016), *in vivo*, threshold: used point of first derivative which exceeded 20 V s^− 1^.

b Yu et al. (2016), *in vivo,* threshold: used point of first derivative which exceeded three times the average first derivative.

c Wilent and Contreras (2005), *in vivo,* threshold: used peak of second derivative, values were averaged from the data for preferred direction and non-preferred direction.

d Mehaffey et al. (2008), *in vitro*, threshold: used point of first derivative which was eight times greater than SD.

e Berman and Maler (1998), *in vitro*.

f Li et al. (2013), *in vitro*, threshold: used point of first derivative which exceeded 10 V s^− 1^.

**Table 3.** Statistical table

	Data structure		Type of statistical test	Power
a	Difference between the average variances of the RMP	Control vs kynurenic acid	Paired *t* test	*p* = 0.0383
b	Difference between the average RMP	Control vs kynurenic acid	Paired *t* test	n.s. (*p* = 0.7372)
c	Difference between the input resistance (hyperpolarizing vs depolarizing)	Control	Paired *t* test	*p* = 3.3 × 10^–12^
d		TTX	Paired *t* test	*p* = 9.9 × 10^–6^
e		QX-314	Paired *t* test	*p* = 2.3 × 10^–4^
f	Difference between the input resistance for hyperpolarizing current injections	Control vs TTX	One-way ANOVA	n.s. (*p* = 0.32)
g		Control vs QX-314	One-way ANOVA	*p* = 5.9 × 10^–5^
h	Difference between the input resistance for depolarizing current injections	Control vs TTX	One-way ANOVA	n.s. (*p* = 0.42)
i		Control vs QX-314	One-way ANOVA	n.s. (*p* = 0.07)
j	Difference in AHP amplitude	Control vs UCL1684	Two-sample *t* test	*p* = 0.0003
k		Control vs EBIO	Two-sample *t* test	*p* = 0.001
l	Difference in AHP area under the curve	Control vs UCL1684	Two-sample *t* test	*p* = 0.0002
m		Control vs EBIO	Two-sample *t* test	*p* = 0.00002
n	Difference in DL neuron current-evoked spiking rate	Control vs UCL1684	Two-way ANOVA	*p* = 0.0013
o		Control vs EBIO	Two-way ANOVA	*p* = 0.000092
p		Control vs BAPTA	Two-way ANOVA	*p* = 1.5 × 10^–15^
q		UCL1684 vs BAPTA	Two-way ANOVA	*p* = 0.00063
r	Difference in DL neuron current-evoked spike height	Control vs BAPTA	Two-way ANOVA	*p* = 2.1 × 10^–12^
s		UCL1684 vs BAPTA	Two-way ANOVA	1.6 × 10^–6^
t		Control vs UCL1684	Two-way ANOVA	n.s. (*p* = 0.14)
u	Difference in DL neuron current-evoked spike width	Control vs BAPTA	Two-way ANOVA	*p* = 1.3 × 10^–31^
v		UCL1684 vs BAPTA	Two-way ANOVA	*p* = 3.7 × 10^–10^
w		Control vs UCL1684 (first 3 spikes only)	Two-way ANOVA	n.s. (*p* = 0.24)
x	AHP reduction between first and second spike	Non-burst spike pairs	One sample *t* test	*p* = 4.45 × 10^–27^
y		Initial burst spike pairs	One sample *t* test	*p* = 1.26 × 10^–13^
z	Spike threshold increase between first and second spike	Non-burst spike pair	One sample *t* test	*p* = 1.24 × 10^–22^
aa		Initial burst spike pairs	One sample *t* test	*p* = 8.30 × 10^–28^

n.s. = not significant.

Hippocampal neurons such as DG granule cells are nearly silent at rest, and discharge very sparsely in response to the animal’s spatial location, i.e., place field (Diamantaki et al., 2016). The low excitability in mature granule cells was shown to be partly due to the constitutive activity of GIRK channels (Gonzalez et al., 2018). We hypothesize that a similar mechanism is contributing to the low RMP of DL neurons in fish, which may partly explain why the difference between RMP and spike threshold is nearly identical in DL and DG cells (Table 2).

With the above examples in mind, we hypothesize that the key biophysical signatures of sparse coding is, for all neurons, a large gap between the RMP and the spike threshold. We further hypothesize that DL neurons will sparsely encode the spatial relations required for memory guided navigation.

### Can the DL network transform PG sequential encounter time stamps to a spatial map?

Previous studies have investigated electrosensory spatial learning in a related gymnotiform fish (Jun et al., 2016; Fotowat et al., 2019). Jun et al., showed that these fish can locate food relative to landmarks in the dark because, after learning, they rapidly navigated to the remembered food location during probe trials (no food). Fotowat et al. (2019) showed that neurons within DD, which has strong reciprocal connections with DL (Giassi et al., 2012a; Elliott et al., 2017), discharged when the fish was engaged in active sensing movements near landmarks. Together, these studies imply that DL is engaged in learning and storing the spatial memories of the relative location of landmarks and food. The electrosense is very local and, for most of their trajectory, the fish had no external sensory cues (Jun et al., 2016). This led Jun et al. to argue that, after leaving a landmark, the fish used the path integration of speed and orientation signals to continuously update its current location and thus compute the trajectory to the remembered food location. Path integration information was assumed to potentially derive from lateral line receptors, vestibular afferents, proprioceptors and vestibular afferents. Bastian (1995) has previously reported that there are brainstem proprioceptive neurons in the gymnotiform fish that are capable of signaling tail bending. Recently, Wallach et al. (2018) found PG neurons are responsive to continuous lateral line input, confirming a second potential source of information related to the fish’s speed. A recent study in the larval zebrafish has demonstrated that vestibular input can evoked strong and widespread activity in the telencephalon that, from the images presented, likely includes DL (Favre-Bulle et al., 2018). We now hypothesize that an encounter with a landmark triggers an autonomous “moving bump” in the DL recurrent network and this is the primary driver for the fish’s estimation of its changing location during its landmark-to-food trajectories. While proprioceptive, lateral line and vestibular input are important, we now hypothesize they merely modulate the essential intrinsic DL network dynamics. We elaborate on this hypothesis below.

In gymnotiform fish, PG cells respond to object motion (electrosensory and visual; Wallach et al., 2018). Anatomic studies indicate that these responses are driven by tectal input (Giassi et al., 2011). The gymnotiform tectum maintains a topographic representation of electrosensory input and tracks continuous object motion (Bastian, 1982). PG neurons generate a major transformation of their tectal input, the majority of PG motion sensitive cells lose topographic information and respond over the fish’s entire body but only to object motion start (all cells) and stop (some cells) and not the intervening continuous motion (Wallach et al., 2018). Wallach et al. proposed that, during navigation in the dark, these PG cells will respond transiently when any part of the fish’s body first encounters a landmark (or food), i.e., the response of the cell when the experimenter moves an object toward the fish is equivalent to its response when the fish moves near a landmark or food. Wallach et al. further proposed that the time interval between encounters could be “read out” from the change in second versus first encounter firing rates of a subset of DL cells.

In the following discussion, we borrow extensively from work on “time” and “place” cells in the mammalian hippocampus using, in particular, the very thorough papers of Kraus et al. (2013) and Pastalkova et al. (2008) as well as the related theoretical papers of Itskov et al. (2011) and Rajan et al. (2016). Kraus et al. (2013) describe hippocampal neurons that respond at specific times during a rat’s motion on a treadmill. These experiments carefully dissociated time from place so that the authors were able to demonstrate the existence of time cells, traditional place cells as well as cells with information on both the time and distance traveled. Pastalkova et al. (2008) and Itskov et al. (2011) had previously argued that sequential activation of cell assemblies is internally generated by hippocampal dynamics and can give rise to time cells independent of sensory input. Kraus et al. (2013) extended this hypothesis and argued that their time cells were driven by both internal network dynamics and external cues such as treadmill speed.

The theoretical papers of Itskov et al. (2011) and Rajan et al. (2016) asked: how might the intrinsic activity of a neural network result in the sequential activation of neuron assemblies, e.g., time cells? Both papers started with the same core architecture, a local excitatory recurrent network that, once activated, was capable of sustained discharge. This is the “bump attractor” hypothesis originally formulated to explain the sustained activity of neurons during a working memory task (Wang, 1999; Wimmer et al., 2014). The theoretical analysis of Wang (1999) demonstrated that slow excitatory synapses, i.e., mediated by NMDA receptors, were required for bump dynamics. Both Itskov et al. (2011) and Rajan et al. (2016) generated “cell assembly sequences” by destabilizing the bump attractor dynamics. Itskov et al. (2011) accomplished this by introducing spike threshold adaptation with a long recovery time constant. In contrast, Rajan et al. (2016) destabilized the bump by introducing asymmetries in synaptic strengths within the attractor so that the attractor dynamics would generate a sequential activation of the cell assembly; a process which necessitated both recurrent connections and external input. In both cases, sequential activation of neurons within the cell assembly are able to produce time cells or other sequential outputs. A recent paper (Heys and Dombeck, 2018) has also suggested that time cells of the entorhinal cortex might be generated by moving bumps in entorhinal recurrent attractor network (Zutshi et al., 2018). This paper did not, however, explicitly discuss the mechanism by which the putative “bumps” would move.

Our earlier work (Trinh et al., 2016) demonstrated that DL contains excitatory local recurrent networks; our earlier work had already demonstrated that DL is highly enriched in NMDA receptors (Harvey-Girard et al., 2007). Trinh et al. (2016), therefore, hypothesized that the DL recurrent network supported bump attractor dynamics capable of memory storage. Our noisy cells suggest that the recurrent connections within DL are, in fact, capable of supporting autonomous discharge. We have now demonstrated that DL neurons exhibit the same threshold adaptation used in the Itskov et al. (2011) model, thus suggesting that the putative DL bumps may not be stable attractors. We have not yet studied the properties of either PG-derived or intrinsic synapses in DL and therefore cannot evaluate whether Rajan et al. (2016)’s architecture might apply. In accordance with the Itskov model, we hypothesize that DL contains unstable bump attractor neural networks that are capable of supporting autonomous sequential activation and thus DL time cells. We assume that, when the fish initially encounters a landmark, the resulting electrosensory-evoked transient discharge in a subset of PG neurons triggers activity in a small region of DL (Giassi et al., 2012b). This activity will then propagate through a subset of the DL network forming a cell assembly temporal sequence (time cells). Following Kraus et al. (2013), we further hypothesize that the sequential activity in this network is modified by ongoing self-motion sensory input, the vestibular, lateral line and proprioceptive input mentioned above. These inputs provide the path integration signals that converts the time cell sequence to a location cell sequence. In functional terms, we propose that the propagation of neural activity in the DL network represents the fish’s estimate of where it is located along the trajectory between a landmark and food. When the fish reaches the food (or another landmark), PG neurons would again discharge to signal the total time/distance traveled (Wallach et al., 2018) and the potential start of a new trajectory. In this model, learning a trajectory from a particular landmark to food would consist of strengthening the synaptic connections of the moving bump induced by that landmark so as to represent the time/location sequence leading from the landmark to food. Such strengthening might result in a Rajan et al. (2016) type mechanism in which directed bump movement was now also a consequence of asymmetric synaptic strengthening.

Our hypotheses are at the moment not testable, because testing would require population recording from or visualizing activity across a large portion of the DL network. What is needed is a teleost that is transparent when adult, whose neurons express a genetically encoded calcium indicator (e.g., gCamp6) and whose pallium might be activated by ethologically relevant transient signals. Fortunately, such a model system has recently become available (Schulze et al., 2018) and may permit direct tests of our hypotheses.

## Synthesis

Reviewing Editor: Katalin Toth, Universite Laval Decisions are customarily a result of the Reviewing Editor and the peer reviewers coming together and discussing their recommendations until a consensus is reached. When revisions are invited, a fact-based synthesis statement explaining their decision and outlining what is needed to prepare a revision will be listed below. The following reviewer(s) agreed to reveal their identity: NONE.

Both reviewers found the study important and well executed. The study provides solid and novel evidence on the biophysical properties of firing adaptation in the fish dorsal lateral pallial neurones. This has important implications for the broader understanding of the evolutionary development of spatial memory formation. Both reviewers raised several points that could help to improve the manuscript. All of the suggestions are constructive and could help to support the points the authors make. In term of new experiments, Revier #3 rightly points out that n=2 is rather low, the authors should increase the 'n' in this set of experiments of explain why such a low 'n' is acceptable in this case. The suggestion on the specific GIRK blocker is great, however it would require significant amount of new experiments and it is not strictly necessary for the validity of the authors' conclusions. The authors should address all other points raised by the two reviewers which can be achieved without completing new experiments.

Rev.#1.

The paper represents a study of the spike generating mechanisms, physiology, and distribution of neuronal cell types in the dorsal lateral pallium of fish, a hippocampal analogue responsible for spatial learning. The authors compare species with either visual (goldfish) or electrosensory (electric fish) spatial navigation, and find similar cell types in both species, suggesting a conservation of the fundamental underlying computations. They extend on this to show that these neurons have firing dynamics expected of neurons encoding a spatial environment sparsely, as has been observed, and predicted by theoretical models of spatial localization. While not examining the currents directly, the authors provide compelling evidence in many cases of the channels underlying the firing dynamics of these cells (although may need to weaken their conclusions in a few cases). Briefly, the authors describe a low resting potential (suggested to be due to GIRK channels, whose presence is confirmed by RT-PCR), spike adaptation (due to SK channels, confirmed pharmacologically, and most likely sodium inactivation, although the evidence is less clear).

Overall, the physiology is compelling, both the basic characterization of the DL neurons, and the contribution of specific channels to aspects of the spike waveform and firing properties.

Finally, the authors present their work in the framework of an attractor network capable of sparse encoding of relative, and sequential points in space. Although speculative, this provides a link to the broader literature, and suggests an interesting way of going forward. While, e.g. some recent discussion has been made about comparative properties of the hippocampus across species, including 'higher' species (see, e.g. the recent comment by Quiroga in the Mar 29 issues of Science), the comparative study of how spatial memory is formed, the ethological specializations within species, and the conserved aspects across species, are all vital to an understanding of the function of this well-studied, but poorly understood part of the brain.

Major comments:

“Noisy” vs. “Quiet” cells- a main point is that the “Quiet” cells, which dominate the data set, have the requisite characteristics to be sparse coders, although the 'noisy' cells are used as a demonstration of recurrency of the network (l. 721). As DL his highly recurrent, how are the 'quiet' cells never noisy? It's unclear if the authors believe these two be two cell classes, or whether (as would be consistent with using the two populations to justify different requirements for the network) a single cell class somehow in a distinct state? Some clarity on this would be helpful.

l. 491 - The authors do not directly test adaptation- it may be more accurate to say gain, or responsiveness, or to directly measure the adaptation in spike rate.

l. 516-519, This is highly speculative, and not directly tested.

l. 563 - Hundreds of milliseconds seems long for Na channel recovery. Are their biophysical papers demonstrating recovery of spike generating Na+ channels at this time scale? The argument that they can be replicated by the model would be strengthened by either citations to support this time scale of recovery from inactivation, or a more thorough discussion of other possible mechanisms such as accumulation of K+ channels. While in practice, it may not matter how the cell achieves these dynamics, if the authors are going to state strongly that this is Na+ inactivation, it should be better supported. For example, does this discussion of spike threshold fatigue starting at l. 614 require that Na+ inactivation be the mechanism?

Minor comments:

l. 75, 82

Many of these studies are in so-called 'pulse' type fish- is there a reason to think that there should be similar encoding in a 'wave-type' fish that is less of an 'active' sensor?

l. 129, Agarose is a gel, and does not polymerize.

L 123/146- are different cutting solutions used for goldfish? Or just different ACSF? It may be easier if the ACSF is all together in the slice preparation part of the methods.

l. 152/153 - “Molecular Devices”

l. 184 - was any attempt made to maintain osmolarity of the intracellular solution? Or was the BAPTA solution 10mM higher in osmolarity? Did that affect cell health?

l. 208 - Do the authors mean here that some cells required a holding current in order to prevent firing or other activity? If so, how are they distinguished from 'noisy' neurons described later?

l. 267/268 - Many things could account for high threshold, it's a little strong to presume that axonal properties are key.

l. 603-604 - I'd assume that hyperpolarization by GIRK channels can lead to any number of possible values for the amount of hyperpolarization it drives, depending on input resistance and the number of channels expressed. 8mV seems overly specific.

l. 662 - “previously reported, in gymnotiform fish on brain stem proprioceptive” is awkwardly worded.

Fig. 9Ai, the lettering appears 'squeezed' and should be correted.

Rev.#2.

This manuscript studied the intrinsic physiology of fish dorsal lateral pallial neurons, especially the cellular mechanisms of firing adaptation. The authors showed the DL neurons have hyperpolarized resting membrane potential and dynamic spike properties (threshold increases following spikes). Overall, the data are of high quality and the findings are interesting. However, there are several concerns need to be addressed.

This manuscript studied the intrinsic physiology of fish dorsal lateral pallial neurons, especially the cellular mechanisms of firing adaptation. The authors showed the DL neurons have hyperpolarized resting membrane potential and dynamic spike properties (threshold increases following spikes). Overall, the data are of high quality and the findings are interesting. However, there are several concerns need to be addressed.

1) The manuscript tried to argue that hyperpolarized resting membrane potential is caused by the activation of GIRK channels and provided the RT-PCR evidence. It would be more convincing if the manuscript can isolate Kir current or with more specific pharmacology (such as GIRK blocker or G-protein blocker) to approve it. Also, the manuscript declaimed that the DL neurons have hyperpolarized resting membrane potential. However, the resting membrane potential of many mammalian neurons are more hyperpolarized.

2) The manuscript showed that SK contribute to the AHP. When look at Figure 6 closely, one can notice that UCL1687 depolarized, while EBIO hyperpolarized the RMP. It should be important to analyze this data. Indeed, it has been shown that SK channels can regulate RMP in mammalian neurons (for example: https://www.ncbi.nlm.nih.gov/pubmed/28982705).

3) In P17 showed that kynurenic acid (10 mM) completely blocked the membrane potential fluctuations of the “noisy” cell. This is a very important evidence to show the membrane fluctuations were caused by glutamatergic synaptic inputs. Also, it suggests that the summation of spontaneous fluctuations is mostly temporal summation of EPSPs. The authors should exam more cells (n =2 now) and present this data in a new figure.

4) The introduction is not very relevant to the main results, the title is somehow misleading, and some of the discussion is not based on the evidence.

Minors: Line 603, GIRK contributes to RMP in many neurons and 8 mV hyperpolarization was seen in a specific neuron (hippocampal neurons). Line 1109, BAPTA is 10 mM, not 10uM.

## Author Response

Authors' response to the editor and reviewers

Editor:

The extended data figures are now referenced in their respective figure legends.

Reviewer #1:

Major comments:

“Noisy” vs. “Quiet” cells- a main point is that the “Quiet” cells, which dominate the data set, have the requisite characteristics to be sparse coders, although the 'noisy' cells are used as a demonstration of recurrency of the network (l. 721). As DL his highly recurrent, how are the 'quiet' cells never noisy? It's unclear if the authors believe these two be two cell classes, or whether (as would be consistent with using the two populations to justify different requirements for the network) a single cell class somehow in a distinct state? Some clarity on this would be helpful.

Considering that we have analyzed both “quiet” and “noisy” cell populations separately throughout this study, it is very difficult for us to drawn any proper conclusions as to whether “quiet” and “noisy” cells originate from two distinct populations or from the same population of cells. Giassi et al. (2012) has presented anatomical evidence suggesting that there may be distinct populations of DL cells. It is therefore possible that noisy and quiet cells are distinct populations. However, it is also possible that some slices contain unhealthy cells whose discharge drives the noisy cells. Given that we cannot distinguish these possibilities, we would prefer to not discuss this point. If the Editor feels that this point needs elaboration, we can easily add a few sentences citing Giassi et al. and mentioning the two possible explanations for the existence of noisy and quiet cells.

l. 491 - The authors do not directly test adaptation- it may be more accurate to say gain, or responsiveness, or to directly measure the adaptation in spike rate.

Lines: 508-509: Yes, we agreed with the reviewer that we did not directly estimate 'adaptation'. Given that DL neurons have a very sparse spike rate, it would be more difficult to obtain a meaningful spike rate. As such, we have replaced the wording of the sentence as per the reviewer's suggestion. We now use 'responsiveness' instead of 'adaptation'.

l. 516-519, This is highly speculative, and not directly tested.

Line 527: Indeed, we agreed with the reviewer that the attribution of the increase in spike width in the UCL1684 condition to the inactivation of SK channels is very speculative and is not essential to the main result which we wanted to highlight (the increase in spike width caused by the addition of BAPTA). As such, we have opted to remove this statement from the text.

l. 563 - Hundreds of milliseconds seems long for Na channel recovery. Are their biophysical papers demonstrating recovery of spike generating Na+ channels at this time scale? The argument that they can be replicated by the model would be strengthened by either citations to support this time scale of recovery from inactivation, or a more thorough discussion of other possible mechanisms such as accumulation of K+ channels. While in practice, it may not matter how the cell achieves these dynamics, if the authors are going to state strongly that this is Na+ inactivation, it should be better supported. For example, does this discussion of spike threshold fatigue starting at l. 614 require that Na+ inactivation be the mechanism?

Lines 569-572 and lines 634-637: We thank the reviewer for this suggestion and we also believe that the argument can be strengthened by adding a few citations to support the long timescale of recovery from Na+ channel inactivation as suggested by the reviewer. As such we have added an introductory sentence in the second paragraph of the spike threshold adaptation section in the results and we have expanded the discussion to better illustrate that the presence of slow Na+ channel inactivation will most likely impact the excitability of DL neurons.

Minor comments:

l. 75, 82

Many of these studies are in so-called 'pulse' type fish- is there a reason to think that there should be similar encoding in a 'wave-type' fish that is less of an 'active' sensor?

Although, the pulse-type specie will increase its electric organ discharge (EOD) near landmarks (Jun et al., 2016) while the wave-type will maintain a constant EOD (Nelson and MacIver, 1999), they will still exhibit similar behaviors when scanning for salient landmarks, notably they will both perform back scanning movements (Jun et al., 2016, Nelson and MacIver, 1999). Considering that the electrosensory circuitry and the midbrain projections to the telencephalon is nearly identical in both species (Lanno and Maler, 1990; Giassi et al., 2012) we expect that the same type of information will be transmitted to the DL region of the telencephalon. By extension, we believe that similar spatial encoding mechanisms will be present in both fish species.

l. 129, Agarose is a gel, and does not polymerize.

Line 128: We agreed with the reviewer, and the sentence has been changed to reflect the solidification of the agarose.

L 123/146- are different cutting solutions used for goldfish? Or just different ACSF? It may be easier if the ACSF is all together in the slice preparation part of the methods.

Lines 130-132 and lines 146-147: Indeed, we have used two different ACSF for both cutting and recording (one per specie). As such, we have combine the two ACSF recipes into the same paragraph as per the reviewer's suggestion.

l. 152/153 - “Molecular Devices”

Lines 151-153: The name of the manufacturer has been corrected as per the reviewer's comment.

l. 184 - was any attempt made to maintain osmolarity of the intracellular solution? Or was the BAPTA solution 10mM higher in osmolarity? Did that affect cell health?

Lines 187-188: Indeed, the osmolality of the BAPTA intracellular solution was maintained, otherwise the cell's health would quickly deteriorate. A sentence was added to better emphasize this point.

l. 208 - Do the authors mean here that some cells required a holding current in order to prevent firing or other activity? If so, how are they distinguished from 'noisy' neurons described later?

Lines 210-211: We appreciate the reviewer's concern that the use of a holding may suggest that the cells are initially “noisy”. However, as stated by the following sentence (lines 210-211), all the cells that were included in the analysis of the resting membrane potential did not require any holding current. A typo was initially present which may have confused the reviewer, but has now been corrected. The figure in the following sentence should have been Figure 2: “For the analysis of the resting membrane potential (Figure 1), only cells...” Furthermore, Fig. 2E suggest that the noise from the noisy cells seems to be independent of the current that is injected to the cell since it still present in both the depolarizing and hyperpolarizing responses. As such, the noisy cells seems to be a subpopulation of DL neurons that are initially noisy with or without the addition of a holding current.

For subsequent analysis (Fig. 3 and onwards), the addition of the holding current was used to keep all of the cells at the same resting membrane potential since the starting membrane potential for each cell can vary a lot according to Fig. 2D and this may bias some of our subsequent analysis.

l. 267/268 - Many things could account for high threshold, it's a little strong to presume that axonal properties are key.

Lines 275-276: We agree with the reviewer's opinion that axonal properties alone may not be the only possible biophysical characteristic that would explain the spike high threshold in DL neurons. However, we wanted to emphasize that the presence of thin unmyelinated axons may suggest that DL neurons have a lower density of Na+ channels than other neurons which in turn may explain the high spike threshold. As per the reviewer's suggestion, we have expanded the sentence to further emphasize this idea.

l. 603-604 - I'd assume that hyperpolarization by GIRK channels can lead to any number of possible values for the amount of hyperpolarization it drives, depending on input resistance and the number of channels expressed. 8mV seems overly specific.

Line 621: We agreed with the reviewer that depending on the cell type, the amount of GIRK channels can lead to a variety in the amount of hyperpolarization driven. The 8mV value was found by Luscher et al. (1997, Neuron) when they compared the resting membrane potential of wild-type CA1 pyramidal neurons with those of GIRK deficient CA1 pyramidal neurons. This was later referenced by the Luscher et al. (2010) review. Since DL neurons are hippocampal-like cells, we thought that this comparison to CA1 hippocampal neurons may provide a slightly more fair comparison. To make this clear for readers, we have specified the cell type in the sentence: “GIRKs can hyperpolarize mammalian CA1 hippocampal neurons by at least 8 mV...”

l. 662 - “previously reported, in gymnotiform fish on brain stem proprioceptive” is awkwardly worded.

Line 689-690: As per the reviewer's suggestion, the sentence has been reworked to make it easier to read.

Fig. 9Ai, the lettering appears 'squeezed' and should be corrected.

As per the reviewer's feedback, the lettering in Fig. 9Ai has been fixed to make it more legible.

Reviewer #2.

Major Comments:

1) The manuscript tried to argue that hyperpolarized resting membrane potential is caused by the activation of GIRK channels and provided the RT-PCR evidence. It would be more convincing if the manuscript can isolate Kir current or with more specific pharmacology (such as GIRK blocker or G-protein blocker) to approve it. Also, the manuscript declaimed that the DL neurons have hyperpolarized resting membrane potential. However, the resting membrane potential of many mammalian neurons are more hyperpolarized.

We thank the reviewer for this in-depth suggestion, however we believe that the difficulties in adopting this suggestion will be very hard to overcome for the scope of this study. For instance, we are unsure that the protein structure of the teleost's version of the GIRK channel will be sufficiently similar as that of the mammalian GIRK. As such, the efficacy of the pharmacological blockers for GIRK channels that are commonly used in mammalian systems will be questioned. We therefore feel that this potentially useful avenue of research is beyond the scope our study.

2) The manuscript showed that SK contribute to the AHP. When look at Figure 6 closely, one can notice that UCL1687 depolarized, while EBIO hyperpolarized the RMP. It should be important to analyze this data. Indeed, it has been shown that SK channels can regulate RMP in mammalian neurons (for example: https://www.ncbi.nlm.nih.gov/pubmed/28982705).

We would like thank the reviewer for this thoughtful suggestion and we have also analyzed this data as per the reviewer's suggestion. We have found that the average RMP for the control (N = 13 cells) was -78.4 {plus minus} 0.8mV while the average RMP for the UCL (N = 7 cells) was -75.9 {plus minus} 1.2mV and lastly, the average RMP for EBIO (N = 6 cells) was -79.1mV {plus minus} 0.9mV. The difference between control and UCL was not significant (two sample t-test: p = 0.1093) and the difference between the control and EBIO was also non-significant (two sample t test: p = 0.6141). As such, we have opted not to include this analysis into the main text since the negative result will not ultimately change the conclusions of our narrative.

3) In P17 showed that kynurenic acid (10 mM) completely blocked the membrane potential fluctuations of the “noisy” cell. This is a very important evidence to show the membrane fluctuations were caused by glutamatergic synaptic inputs. Also, it suggests that the summation of spontaneous fluctuations is mostly temporal summation of EPSPs. The authors should exam more cells (n =2 now) and present this data in a new figure.

Line 357-361: We agreed with the reviewer that we may have understated the importance of this experiment and as such, we have added 3 more cells to our analysis of the synaptic noise. Furthermore, we have expanded Fig. 3 of the results to include the results of the kynurenic acid experiments (Fig. 3D). In essence, we have measured the variance of the resting membrane potential before and after the bath application of the 10 mM kynurenic acid and we have shown that the variance decreased to a negligible value after the addition of the broad spectrum synaptic blocker (Fig. 3E). Lastly, we have also showed that the effect of the kynurenic acid was strictly on the synaptic inputs since the resting membrane potential remained unchanged after the addition of the synaptic blocker (Fig. 3F).

Minor comments:

Line 603, GIRK contributes to RMP in many neurons and 8 mV hyperpolarization was seen in a specific neuron (hippocampal neurons).

Line 621: Indeed, we agreed that this sentence may have provided some confusion for the readers. As such, we have specify the cell type to make the sentence clearer.

Line 1109, BAPTA is 10 mM, not 10uM.

Line 1128: We thank the reviewer for noticing this typo mistake. The BAPTA concentration has been changed to 10 mM as intended.

Additions to the Ms not requested by the Editor or Reviewers.

We have updated the Fotowat et al. (2019) reference (instead of 2018) throughout the text.

Methods:

Line 168-171: We have added a small section for the blocking of synaptic noise experiment: “A subset of DL cells exhibited membrane 'noise'. We bath applied the non-selective antagonist, kynurenic acid (10 mM; Millipore Sigma, Canada), to block ionotropic glutamatergic transmission in order to determine whether this noise was due to synaptic input to DL cells.”

Line 212-216: We have added a small description for the analysis of the synaptic noise blocking experiment. “For the analysis of the average RMP (and variance) in Fig.3, a total of 2 minutes of recording (binned into 10s sweeps) were analyzed for each cell before and after the addition of the synaptic blocker (for a total of 4 minutes per cell). If the recording trace contained any spontaneous action potentials, the action potentials were replaced with the membrane potential recorded in the prior 100 ms.”

Results:

Line 355: A typo has been corrected. The synaptic blocker used in Marcoux et al. (2016) was CNQX (for AMPA) and APV (for NMDA) and not kynurenic acid as previously written.

Line 356: Verb was changed to past tense.

Line 359: The reference for lack of recurrent connections in the Apteronotid ELL has been

corrected to Maler L, Sas EK, Rogers J (1981,Journal of Comparative Neurology).

Discussion:

We have expended the discussion as follows:

Lines 641-644:“We note that the AHP and slowly recovering Na+ inactivation have very different effects on neuron excitability (Benda et al., 2010). The dynamical interaction of these biophysical mechanisms (not currently known) will likely be a critical determinant of the spiking response of DL neurons to their time varying synaptic input.”

Lines 680-684: “Fotowat et al. showed that neurons within DD, which has strong reciprocal connections with DL (Giassi et al., 2012a; Elliott et al., 2016), discharged when the fish was engaged in active sensing movements near landmarks. Together, these studies imply that DL is engaged in learning and storing the spatial memories of the relative location of landmarks and food.”

Figures:

Fig.6: The scale bar in Fig. 6A has been corrected (it was originally 200 ms, when it should have been 100 ms).

Line 1113: A typo was found in the figure legend of Fig. 6B: the duration of the example trace was actually 750 ms (and not 1000ms).

Statistical Table (Table 3):

The statistics for the kynurenic acid experiment were added to the table.
