# Erratum: Trinh et. al., Cellular and Network Mechanisms May Generate Sparse Coding of Sequential Object Encounters in Hippocampal-Like Circuits

**DOI:** 10.1523/ENEURO.0327-20.2020

**Published:** 2020-08-31

In the article “Cellular and Network Mechanisms May Generate Sparse Coding of Sequential Object Encounters in Hippocampal-Like Circuits,” by Anh-Tuan Trinh, Stephen E. Clarke, Erik Harvey-Girard, and Leonard Maler, which published online on July 19, 2019, an equation appeared incorrectly because of an author error. In the “Inactivating exponential integrate and fire model (iEIF)” section of the Materials and Methods, Equation 1 should have stated “for *V* < θ” instead of “for *V* < *V_T_*.” The online version has been updated.

