# Erratum: Trinh et al., “Cellular and Network Mechanisms May Generate Sparse Coding of Sequential Object Encounters in Hippocampal-Like Circuits”

**DOI:** 10.1523/ENEURO.0293-21.2021

**Published:** 2021-08-19

In the article “Cellular and Network Mechanisms May Generate Sparse Coding of Sequential Object Encounters in Hippocampal-Like Circuits,” by Anh-Tuan Trinh, Stephen E. Clarke, Erik Harvey-Girard, and Leonard Maler, which published online on July 19, 2019, the legend for Extended Data Figure 8-1 was duplicated from the legend for Extended Data Figure 5-1 because of a production error. The legend should instead read:

"Current-evoked spiking decreases the AHP amplitude of DL neuron. ***A***, Example trace of a DL neuron’s response to a +60 pA current step injection lasting 500 ms. Consecutive spiking causes the AHP amplitude to decrease when compared with the first AHP as emphasized by the arrow. A black dashed line is placed to coincide with the minimum of the first spike’s AHP. We hypothesize that the decrease in AHP amplitude is because of a reduction in Ca^2+^ influx (i.e., Ca^2+^-dependent Ca^2+^ channel inactivation) and subsequent reduction in SK channel opening. ***B***, The decrease in AHP amplitude between the second and first spikes is plotted as a function of the time interval between the first two spikes similar to Figure 8*B*. Each black square represents a spike pair taken from a trace that did not contain a burst (total of 160 nonburst spike pairs), while each gray triangle represents a spike pair taken from a trace that contained a burst at the beginning of the trace (total of 117 burst spike pairs). The majority of the AHPs are reduced throughout the 300 ms test period without any evident recovery trend."

Additionally, Itskov et al. (2008) should not have appeared in the reference list; all reference citations should instead be to Itskov et al. (2011). The online version has been corrected.

