# Supplementary figures and images for: Cellular and Network Mechanisms May Generate Sparse Coding of Sequential Object Encounters in Hippocampal-Like Circuits
Source: eNeuro. 2019 Aug 19;6(4):ENEURO.0108-19.2019. doi: 10.1523/ENEURO.0108-19.2019 (PMC6709220; doi:10.1523/ENEURO.0108-19.2019)

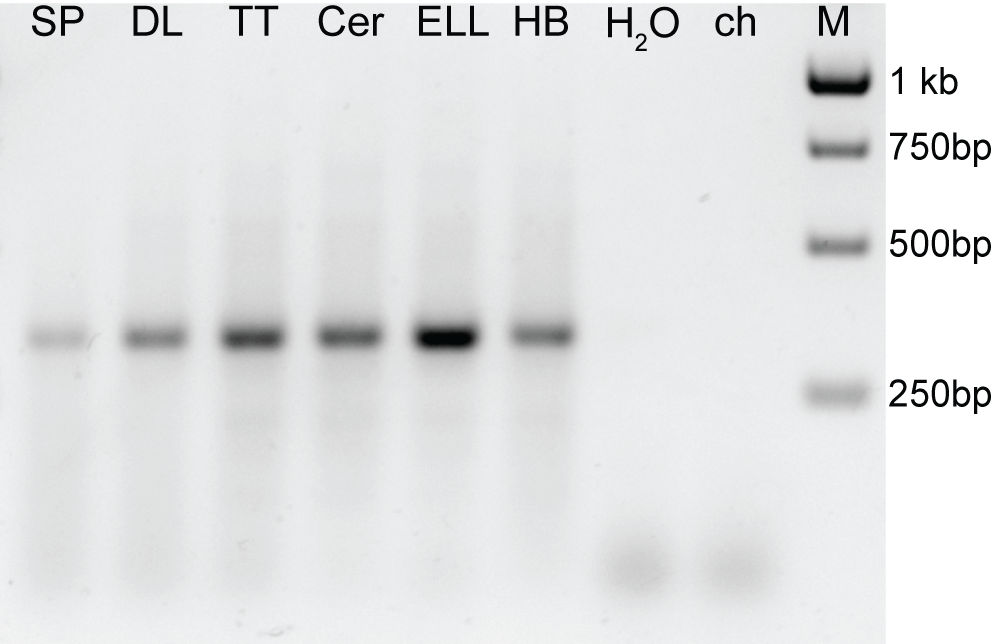

Supplement: Extended Data Figure 5-1 — GIRK channel mRNA expression obtained from RT-PCR in the Apteronotus brain using pan-PCR primer pairs in conserved regions. GIRK channels are ubiquitously expressed albeit at variable levels. In particular they are expressed in DL. SP, subpallium; TT, tectum/torus; Cer, cerebellum; HB, hindbrain; ch, chicken (negative control); M, molecular marker. Download Figure 5-1, TIF file. [file sup_enu-eN-NWR-0108-19-s02.tif]

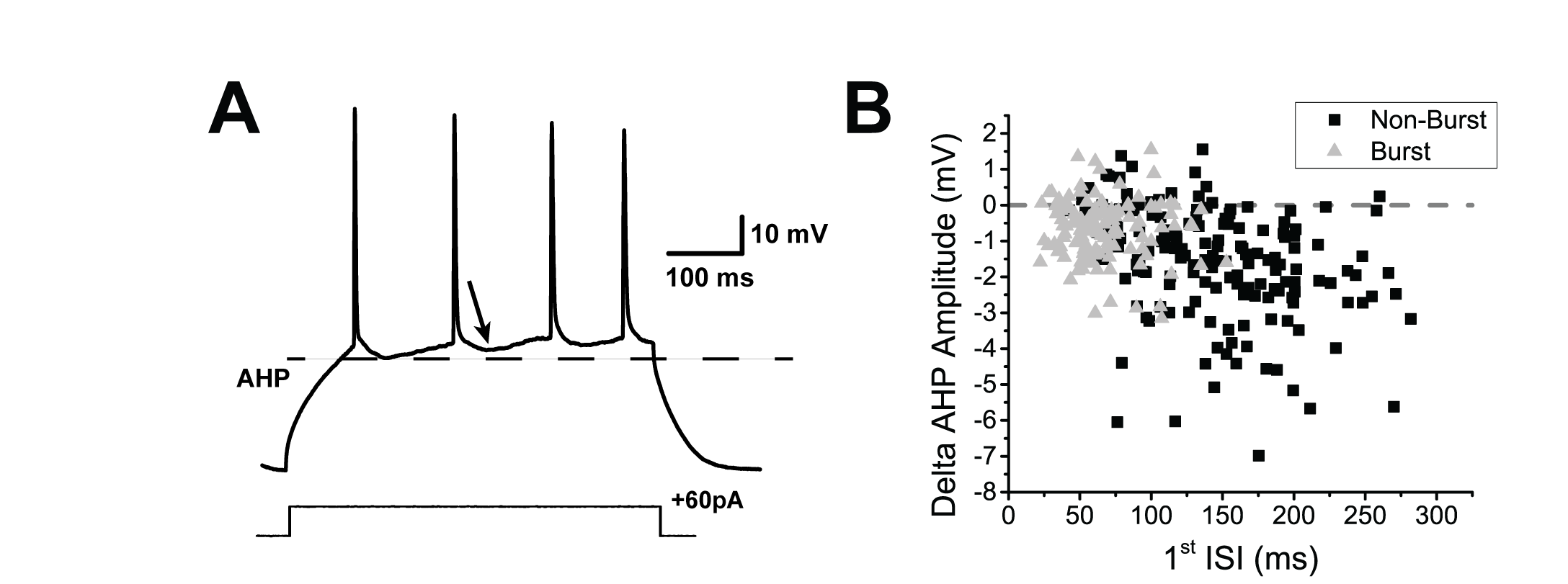

Supplement: Extended Data Figure 8-1 — GIRK channel mRNA expression obtained from RT-PCR in the Apteronotus brain using pan-PCR primer pairs in conserved regions. GIRK channels are ubiquitously expressed albeit at variable levels. In particular they are expressed in DL. SP, subpallium; TT, tectum/torus; Cer, cerebellum; HB, hindbrain; ch, chicken (negative control); M, molecular marker. Download Figure 8-1, TIF file. [file sup_enu-eN-NWR-0108-19-s03.tif]
